# Computational Diagnostic Techniques for Electrocardiogram Signal Analysis

**DOI:** 10.3390/s20216318

**Published:** 2020-11-05

**Authors:** Liping Xie, Zilong Li, Yihan Zhou, Yiliu He, Jiaxin Zhu

**Affiliations:** College of Medicine and Biological Information Engineering, Northeastern University, Shenyang 110169, China; longzilipro@gmail.com (Z.L.); 20185423@stu.neu.edu.cn (Y.Z.); 20185457@stu.neu.edu.cn (Y.H.); 20175563@stu.neu.edu.cn (J.Z.)

**Keywords:** electrocardiogram, classification, feature engineering, deep learning, machine learning

## Abstract

Cardiovascular diseases (CVDs), including asymptomatic myocardial ischemia, angina, myocardial infarction, and ischemic heart failure, are the leading cause of death globally. Early detection and treatment of CVDs significantly contribute to the prevention or delay of cardiovascular death. Electrocardiogram (ECG) records the electrical impulses generated by heart muscles, which reflect regular or irregular beating activity. Computer-aided techniques provide fast and accurate tools to identify CVDs using a patient’s ECG signal, which have achieved great success in recent years. Latest computational diagnostic techniques based on ECG signals for estimating CVDs conditions are summarized here. The procedure of ECG signals analysis is discussed in several subsections, including data preprocessing, feature engineering, classification, and application. In particular, the End-to-End models integrate feature extraction and classification into learning algorithms, which not only greatly simplifies the process of data analysis, but also shows excellent accuracy and robustness. Portable devices enable users to monitor their cardiovascular status at any time, bringing new scenarios as well as challenges to the application of ECG algorithms. Computational diagnostic techniques for ECG signal analysis show great potential for helping health care professionals, and their application in daily life benefits both patients and sub-healthy people.

## 1. Introduction

Cardiovascular disease (CVD) is the leading cause of death in the world, according to the statistics of the World Health Organization. More than 30% of global deaths are caused by cardiovascular disease each year, and more than 130 million adults are estimated to be suffered from cardiovascular disease by 2035 [1]. As a technique of recording electrophysiological signals of heart, electrocardiogram (ECG) with the advantages of non-invasiveness and real time, has been widely used in medical care, such as heartbeat recognition, blood pressure detection, and disease detection [2,3]. Since the first discovery of ECG by Augustus Desire Waller in 1887 [4], ECG signals have been widely used in the diagnosis of heart diseases, such as arrhythmia and myocardial infarction. Computer-assisted medical diagnoses based on ECG signals can give professional suggestions or even make a judgment directly by looking for characteristic patterns on the ECG signals. According to the number of leads, common ECG acquisition device can be divided into 1-lead ECG, 3-lead ECG, 6-lead ECG and 12-lead ECG [5]. 12-lead ECG is the most commonly used type in clinical practice [6], because of its ability to record the potential changes of 12 sets of electrode patches attached to the body in standardized locations at the same time. Compared with other types of ECG acquisition equipment, 12-lead ECG possesses more detailed information about heart activities and is often used in professional diagnosis and treatment in hospitals. With the advancement of software and hardware, single-lead ECGs and photoplethysmography (PPG) sensors which are more readily available, inexpensive, convenient, and easily integrated into portable devices have entered people’s daily life. All these characteristics make it suitable for long-term monitoring and expand the application of ECG.

Analysis and interpretation of the ECG often perform by professional doctors [7], which largely depends on the doctor’s training, certifications experience and knowledge [8]. However, even experts cannot get enough information from the ECG signals [9]. With the advancement of algorithm and physical hardware technology, automated diagnostic systems become to play an increasingly important role in the diagnosis of heart disease, transitioning from selecting potentially effective lesion features for doctors to independent decision making. ECG features are unique information extracted from ECG signals and are used to represent the state of the heart. In addition to morphological features that can be observed, features such as wavelet features and statistical features have also been proven to be effective in diagnosis [10,11,12]. In recent years, the fast development of machine learning methods has brought new opportunities to medical signal analysis [13], making ECG analysis more intelligent and efficient [14]. At present, wearable medical devices in healthcare can even replace 12-lead ECG acquisition devices [15,16], which can detect some common cardiovascular diseases, such as atrial fibrillation, arrhythmia, and stress.

**Figure 1:**
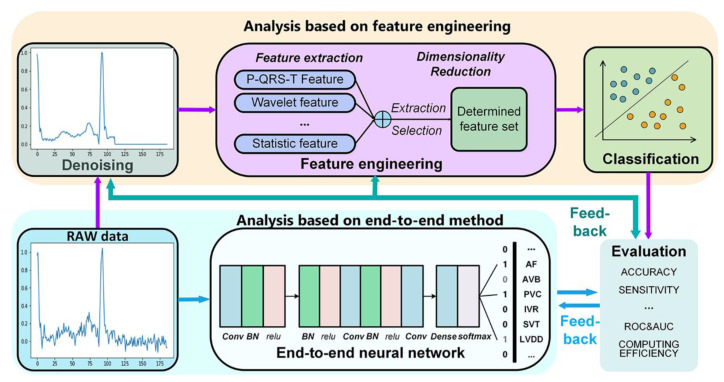
Process of computational diagnostic techniques for electrocardiogram signals.

There are many reviews on ECG in recent years [3], however, most of them focus on single processing techniques, such as ECG pre-processing, feature engineering techniques. Lyon et al. analyzed several commonly used classifier algorithms in ECG analysis, including support vector machine (SVM), random forest algorithm, Bayesian network, and neural network networks [17]. However, there are few systematic reviews on the application of deep learning in ECG analysis. With the enhancement of computing power and the development of cloud computing technology in recent years, machine learning and deep learning have shown great advantages in ECG analysis. More studies choose to use dimensionality reduction methods to extract effective features from a large feature sets, instead of only studying pre-selected specific features. With the development of End-to-End models represented by artificial neural networks, ECG analysis no longer relies on feature engineering. At present, end-to-end models featured by deep neural network’s structure have aroused more and more researchers’ attention. All of the parameters of the end-to-end models are trained jointly, rather than step by step. Without using any hand-crafted techniques, ECG analysis based on end-to-end model has great advantages in accuracy and robustness. This article summarizes the latest computational diagnostic techniques based on ECG signals for estimating CVD conditions (Figure 1). The procedure of ECG signals analysis based on machine learning is discussed from data preprocessing, feature extraction and selection, classification, and application. Notably, End-to-End models based on deep learning algorithms for ECG analysis have been summarized, which enable the analysis process no longer to require a feature extraction with hand-crafted techniques. We also discuss the development trends and challenges of computational diagnostic techniques for ECG analysis, which demonstrates great potential of ECG-assisted analysis in health diagnosis.

## 2. Data Preprocessing

### 2.1. Noise in Electrocardiogram (ECG)

Normal ECG signals are time-varying signals with a small amplitude ranging from 10 μV to 5 mV. Their typical value is 1 mV and their frequencies range from 0.05–100 Hz, mainly concentrated in the 0.05~35 Hz range. Most ECG analysis systems require relatively noiseless ECG signals to achieve accurate and reliable CVDs diagnosis [18]. However, in practice ECG signals are often disturbed by various noises and artifacts, including baseline drift, electrode contact noise, power-line interference (PLI), and electromyographic (EMG) noise, which lead to ECG distortion and affect feature extraction [19,20,21].

Patient movements, poor electrode contact, and changes in electrode skin impedance cause baseline wander and abrupt drift noises. During Holter monitoring, ECG monitoring in moving ambulance or exercise, baseline wander plays a significant role in noises [22]. The frequency range of the motion noise is usually 1–10 Hz. The amplitude of baseline wander caused by respiration varies by ~15% of peak-to-peak ECG amplitude at frequencies ranging from 0.15 Hz to 0.3 Hz [19]. It is noted that the severe baseline wanders or motion artifacts can distort the ST-segment and other low-frequency components of ECG signals. The ST segment distortion may lead to the wrong diagnosis of myocardial infarction, Brugada syndrome, and other ST-segment related abnormalities.

Power-line interference (PLI) (50/60 Hz) is mainly contributed by inductive and capacitive couplings of ubiquitous power lines in the ECG signal acquisition circuitry [20]. The lower frequency noise components of the PLI are mixed with the frequency content of the ECG signal. The severely structured noises can distort the morphological features such as amplitude, duration, and shape of low-amplitude local waves of the ECG signal. In particular, the P-wave distortions can lead to the wrong diagnosis of atrial arrhythmias such as atrial enlargement and fibrillation [23]. Proper shielding, avoiding loose connection in wires and appropriate placement of electrodes can minimize the PLI.

EMG noise is contributed by the electrical activity of muscles during periods of contraction. The frequency distribution of EMG noise is basically within 0 Hz to 500 Hz and mainly concentrates in the range from 50–150 Hz. EMG noise with a high frequency above 100 Hz can be removed by a low-pass filter with an appropriate cut-off frequency. Previous studies demonstrated that EMG artifacts significantly altered the shapes of local waves of the ECG signal because the frequency of the noise was considerably superimposed with that of the ECG signals in the range of 0.05–100 Hz. Thus, removal of muscle artifacts is quite challenging without distorting the clinical features, which is essential for recognizing various ECG arrhythmia [24].

### 2.2. Methods of ECG Denoising

The frequency of the ECG signal ranges from 0.05–100 Hz and mainly concentrates in the range of 0.05~35 Hz. High-frequency noises, such as PLI, EMG, distort both temporal and spectral characteristics of the ECG signal drastically. Generally, it is hard to locate the QRS complex accurately because the EMG and PLI noise usually mask the characteristics of the ECG signal on the higher end of its frequency range. Moreover, the relatively low-frequency noises, such as baseline drift caused by respiration, coincides with the ECG signal in low-frequency. Therefore, the removal of noises without losing the main characteristics of ECG signals is the basis of the subsequent algorithm, which will seriously affect the judgment accuracy of the doctor. Suppressing the interference of both high-frequency and low-frequency noise on the ECG signal and improving the signal-to-noise ratio of the signal becomes an important step in any signal processing techniques and computer-aided diagnosis of heart disease. Most instruments for ECG acquisition decrease noise on ECG signals using hardware high-pass circuits, which are usually used to reduce obvious high-frequency noise such as instrument noise. However, it is difficult to adjust filtering parameters, resulting in limited noise reduction, or even signal distortion. Various denoising algorithms have been developed for ECG denoising, including digital filtering, wavelet transform, and empirical mode decomposition [25].

Digital filters are widely used in signal processing to filter out unwanted parts of the signal, which are uniquely characterized by discrete-time Fourier transform of the time response in the frequency domain. The digital filters are categorized into finite-duration impulse response (FIR) filters and infinite-duration impulse response (IIR). Compared with FIR filters, the filter coefficients of IIR filters can be adjusted by the feedback difference equation, which allows digital filters to perform adaptive filtering. In general, low-pass filters and band-pass filters can filter out obvious noise signals by choosing appropriate parameters. However, the noise reduction effect of the filter with a fixed threshold is very limited due to the large frequency range and various amplitudes of the contaminated ECG signal.

Wavelet transforms are widely used in ECG noise reduction due to their good time-frequency characteristics, coefficient compression characteristics, noise dilution characteristics and removal of redundancy [18]. The wavelet transform does not change the signal’s time characteristics, and the wavelet analysis possesses good local characteristics in both time domain and frequency domain, which continuously decomposes the signal into the detail coefficients of high frequency and the approximate coefficients of low frequency. Since the noise component usually appears in the detail coefficients, the noise signal can be filtered out by threshold quantization of the detail coefficient. Finally, the ECG signal can be obtained by wavelet reconstruction of the high-frequency and low-frequency coefficients. Therefore, the selection of appropriate threshold functions is the key to achieve the desired effect of the noise-filtering effect. The performance of the thresholding strategy depends on the type of threshold method and the threshold rules for a specific application [26]. Hard and soft threshold functions are widely used in ECG denoising [27]. The reconstructed signals obtained by the hard threshold method have better approximation, but may lead to the oscillation of the reconstructed ECG signal, while the reconstructed signals obtained by the soft threshold method have better smoothness but larger error. Han et al. proposed an improved wavelet denoising method named sigmoid function-based thresholding [28], which is a compromising technique between hard and soft thresholding. The improved wavelet thresholding method performs well to some extent in maintaining the amplitudes of the main characteristic peaks. Ata et al. proposed an ECG signal denoising method using a fuzzy threshold and wavelet analysis [29]. They used a loop-based algorithm to search the optimal parameters of the fuzzy s-function, and determined the proper threshold and variance values based on the basic search algorithm for good denoising performance. Compared with soft and hard thresholding methods, the proposed method shows superior performance. Discrete wavelet transform (DWT) has a good denoising performance for high-frequency noise, but it usually results in the loss of key information at low-frequency. Pratik et al. proposed an ECG denoising technique using DWT and non-local mean (NLM) estimation [30]. The noisy ECG signal was decomposed into low- and high-frequency approximation and detail coefficients by two-level DWT decomposition. After that, thresholding the detail coefficients is performed on the two-level detail coefficients to eliminate high-frequency noise. Since the second-level low-frequency coefficients contain most of the ECG signals, the NLM estimation of the second-level approximation coefficients is performed separately to remove low-frequency noise. The method can remove noise in the low-frequency region better and faster. It is superior to other methods when tested on the MIT-BIH arrhythmia database. Besides, the choice of wavelet parameters is challenging, because it is usually performed by experience. Researchers have proposed some effective methods based on parameter adjustment for ECG signals denoising. β-hill climbing method shows an effective improvement to the climbing algorithm, which can build search trajectories in the search space until reaching the local optima [31]. Alyasseri et al. combined β-hill climbing algorithm with wavelet transform for ECG signal denoising [32]. The β-hill climbing is employed to find the optimal wavelet parameters for ECG denoising, which can achieve the minimum mean square error between the original and denoised ECG signals. The proposed method showed an excellent performance in filtering out noise, especially for the ECG signal with low frequency noise, and achieved a high quality of ECG denoising signal, which was favorable for medical diagnosis.

Empirical mode decomposition (EMD) serves as an alternative to wavelet analysis for ECG denoising [33]. The EMD is an adaptive data-driven technique that does not require pre-determination of parameters. It can adaptively decompose signals into different intrinsic mode functions (IMF), which is suitable for analyzing nonlinear and nonstationary time-series signals. The EMD can achieve noise reduction by reconstructing the IMFs of the signals and removing the IMFs of noise [34]. The decomposed IMFs are selected by power or entropy approach. Low-order IMFs mainly capture high-frequency noise such as muscle artifact, power line interference and instrument interference, while high-order IMFs can capture slow oscillation noise such as baseline drift. Usually, the noises are mainly contained in the lower order IMFs, while the useful signal is included in the higher-order IMFs. Therefore, ECG signal can be denoised in the EMD time domain by selection of higher-order IMF signals based on the assumption that noise and signal are separated in frequency bands [25]. Hasan et al. decomposed ECG signal into six order IMFs using EMD, and higher order IMFs were used to form a modified ECG signal. The denoising signal was fed into a CNN architecture that classifies the ECG signals for diagnosis of cardiovascular diseases [35]. The results based on MIT-BIH, St.-Petersberg, PTB databases showed that the classification accuracy of the reconstructed signals was much higher than that of the original signals, and it was superior to other approaches using the neural network as classifiers. EMD has strong adaptability, but its main disadvantage is mode mixing effect, which is defined as a give IMF composed of oscillations of dramatically disparate time scales, or that oscillations with the same time scale are distributed in different IMFs [36]. Ensemble empirical mode decomposition (EEMD) as a truly noise-assisted data analysis method represents a substantial improvement of EMD. The effect of mode mixing is weakened by adding white noise to signal analysis. Chang compared the filtering performance of EEMD, EMD, Butterworth filter and Wiener filter both on normal ECG and arrhythmia ECG [37]. Synthetic noises including 50 Hz PLI, EMG, baseline wander noises were added into simulated and real ECG signals. The IMF spectrum distribution showed that EEMD reduced the mode mixing. The results showed that EEMD not only has lower mean square error under various noise contamination scenarios but also superior on conservation of filtered ECG waveform, especially for arrhythmia ECG. There are many EMD or EEMD-based approaches to ECG denoising generally deal with the PLI, EMG and white noise [38,39], and most of them mainly consider the noise energy in the first order IMF, which will occur the over-cancellation and eventually lead to distortion in the reconstructed ECG signal. Liu et al. proposed a grey spectral noise cancellation (GSNC) scheme using EMD, EEMD and grey spectral noise estimation (GSNE) to deal with the PLI and EMG noises for ECG signals [40]. The proposed GSNC scheme is a two-stage discrimination scheme based on the IMFs’ noise magnitude spectrum. The EMD decomposed the input ECG signals into IMFs in the first stage and the EEMD reconstructed and decomposed the noisy IMFs in the second stage. The GSNE and noise magnitude spectrum were used in both stages to estimate noise in IMFs and calculated the related noise energies. The results showed that the GSNC scheme was superior to EMD and EEMD methods when tested on the MIT-BIH database where different signal-noise-ratio levels for the PLI and EMG noise were considered.

## 3. Feature Engineering

ECG reflects the regular or irregular beating activity of heart because it records electrical impulses generated by heart muscles. Therefore, it is crucial to extract as much meaningful clinical information as possible from ECG signals. Doctors often make diagnoses by observing the morphological characteristics of P-QRS-T waves, which largely depends on doctors’ experience and usually takes a long time. Computer-aided analysis based on feature engineering has greatly improved the efficiency and accuracy in ECG analysis. ECG signals consisting of abundant data points can be extracted a small number of features in terms of its peak amplitude, morphology, energy and entropy distribution, frequency content, intervals between events, which can represent the behavior of the ECG signal. These features commonly applied to ECG diagnosis include P-QRS-T features, statistical features, morphological features, frequency-domain features and other more complex parameters, which provide effective tools for doctors’ judgment. Advanced algorithms extract features according to the needs of the task and automatically selects specific features to achieve precise diagnosis. As the core of ECG analysis, feature extraction and selection play a decisive role in the performance of the algorithm.

### 3.1. Features for Disease Diagnosis

#### 3.1.1. P-QRS-T Complex Feature

**Figure 2:**
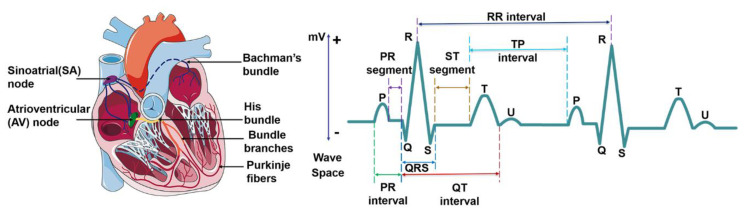
Cardiac electrical conduction system and the electrocardiogram signal.

ECG waveform reflects the activity of heart tissue, which is a very weak physiological low-frequency electrical signal. The maximum amplitude is no more than 5 mv and signal frequency is in the ranges from 0.05–100 Hz. Normal ECG waveform consists of P-wave, QRS complex, T-wave and sometimes U waves (Figure 2). Morphological features of ECG signal include different peak amplitudes, peak intervals and QRS complex, etc. The typical morphological features of ECG signal are summarized in Table 1. Sinoatrial node (SA) depolarization occurs before the depolarization of atrial myocytes, so it is before P-waveform. But SA is inside the heart, and its electrical activity is difficult to be collected on the body surface. The excitement of the SA is transmitted to the right atrium, and then to the left atrium via the ventricular tract, forming a P-wave which represents the excitement of the two atria. P-waveform is relatively small, with a round shape, an amplitude of about 0.25 mV and a length of 0.08~0.11 s. When atrial enlargement occurs, the conduction between the two atria will be abnormal, resulting in P-mitrale or P-pulmonale waves. P-R interval refers to the interval from the onset of P wave to the beginning of QRS complex on an ECG signal. In normal ECG, P-R interval is 0.12–0.2 s, which corresponds with the spread of the electrical conduction in atrioventricular junction. A prolonged P-R interval reflects impaired atrial conduction, and maybe an indicator of ischemic strokes [41]. The QRS complex represents the spread of a stimulus through the ventricles. A complete QRS complex consists of Q-, R- and S-wave. R wave is long and narrow, representing the depolarization of the left ventricle apex [42]. The typical duration of QRS complex is about 0.06–0.1 s. Heart rate is usually measured by recording the number of QRS complexes in a minute. The comprehensive electric field vector of ventricular myocytes changes many times in the process of excitation, which forms the signal with multiple changes in size and direction. When the conduction block of the left and right bundle branches of the heart, ventricular enlargement or hypertrophy occurs, the QRS complex will widen, deform and prolong. T wave follows QRS complex, and is produced by repolarization of ventricular myocytes with an amplitude of 0.1–0.8 mV and lasts for 0.05–0.25 s. The T wave is always positive and is useful for the diagnosis of certain cardiovascular diseases. It’s common to detect abnormal T waves (e.g., inverted T waves) by chest leads on patients with pulmonary embolism (PE). Marcinkevics. R. et al. found that the T-wave amplitude of patients with arrhythmia right ventricular dysplasia (ARVD) was significantly different from that of normal patients [43]. U-wave is the last unsteady and smallest wave in the ECG, which shows a circular upward deflection. Sometimes, U-wave may not be observed because of its small size. The formation of U wave is controversial. Generally, U-wave is thought to represent repolarization of the Purkinje fibers. Usually, U wave has the same polarity as T wave. In clinical diagnosis, transient U-wave inversion can be caused by local myocardial ischemia or hypertension [44].

Segments and intervals of the ECG signal reflect each stage and cardiac cycle of heart contraction, which should be completed within a specific period for a healthy person. The abnormal period indicates something wrong with the heart [55]. R-R interval is the time elapsed between two consecutive R waves of the QRS signal. It is usually employed to assess ventricular rate. During sinus rhythm, patients with short-term risk of paroxysmal atrial fibrillation (PAF) tend to show higher RR interval variability [45]. Q-T interval refers to the time from the beginning of QRS to the end of T wave, representing the total time required for ventricular depolarization and repolarization. It is worth noticing that P-R interval is different from the P-R segment, which is the time between the end of the P-wave and the beginning of the QRS wave. The P-R interval reflects the time delay between atrial and ventricular activation, which indicates whether impulse conduction from the atria to the ventricles is normal. The proper feature selection of ECG waveform can be used in some specific disease diagnosis. S-T segment refers to the period between the end of QRS complex and the start of T wave. During the S-T segment, the myocytes of the left and right ventricles are in the excitation period, so the contribution of the combined electric field vector formed by the two is very small in the body surface ECG, and the signal in S-T segment is at the baseline level. When a segment of myocardium is ischemic or necrotic, the ventricular potential difference still exists after the completion of depolarization, which is manifested as S-T segment shift on the ECG waveform [51]. The typical disease that is associated with S-T segment is the myocardial infarction (MI), which is commonly known as a heart attack, occurs when the blood supply to the portion of the heart is blocked, causing some heart cells to die. The corresponding ECG signal is depicted in the elevated ST segment, increased Q wave amplitude and inverted T wave.

#### 3.1.2. Fourier Transform Feature

Fourier transform (FT) as a well-known tool for investigating a signal in frequency domain, is widely used in signal processing. FT converts a signal from the time domain into the frequency domain, and the amplitude spectrum and phase spectrum of each frequency component can be easily found. Fast Fourier Transform (FFT) greatly reduces the times of multiplication required for computing the discrete Fourier transform and significantly improves the processing speed. Frequency domain analysis can be used to locate feature points for ECG signal analysis. The QRS complex is the most striking waveform within the ECG signal, which serves as the basis for the automated determination of the heart rate. Gothwal et al. identified the peaks in the ECG signal using FFT [56]. Feature as RR interval metrics and heart rate calculated from these feature points were classified by neural networks to identify the diseases. Although FFT provides detailed frequency information, it fails to provide any information regarding the time of occurrence of the frequency components for a non-stationary input signal. This problem can be solved by short-time Fourier transform (STFT). The STFT can be used to convert non-stationary signal from time domain to time-frequency domain using a window function, which is also called windowed Fourier transform. The signals were separated into a series of quasi-stationary parts within the window period. STFT has been explored spectral information that provides time and frequency information simultaneously for a signal varying with time. Many frequency-domain features in medicine are associated with certain diseases. When arrhythmia occurs, QRS complex will change obviously, resulting in abnormal change of high-frequency component. Minami et al. converted each QRS complex to a Fourier spectrum, and then the power spectrum was calculated [57]. The features were fed to neural network algorithm for rhythm classification. The method could discriminate supraventricular rhythms from ventricular ones with high sensitivity and specificity (≥0.98). Energy distribution of the ECG signal can be computed by STFT, the features are then extracted from the energy distribution and fed to classification algorithms for diagnosis. Huang et al. utilized STFT and 2D-CNN for ECG arrhythmia classification [58]. The time-domain signals of ECG were first transformed into time-frequency spectrograms by STFT. Subsequently, the STFT-based spectrograms were fed to a 2D-CNN, resulting in an averaged accuracy of 99.00%. STFT extracts time-frequency information regarding the exact location of frequency components of ECG signal, however, the major drawback of this STFT is that there is a tradeoff between its time and frequency resolutions in STFT mainly due to the fixed window size [59]. A long window results in good frequency resolution and poor time resolution, while short window results in good time resolution and poor frequency resolution. To overcome this drawback, the wavelet transform employs a time-scale resolution scheme for ECG signal analysis.

#### 3.1.3. Wavelet Feature

Wavelet analysis as a powerful signal processing technique for analyzing time series with many different timescales can decompose the signal on a time-scale domain into shifted and scaled versions of the base wavelet [60,61]. Compared with Fourier transform, wavelet transform provides a variable “time-frequency window”, which allows us to change its scale dynamically. The window size will wide at low frequencies region and narrow at high frequencies region, therefore, it is a suitable tool for all frequency ranges, resulting in an improvement of accuracy both in time and frequency domain. Wavelet provides a method for compression or enhancement features [62]. Because of its high resolution of time and frequency, wavelet transform can recognize the abstract and hidden features of ECG signals [63]. There are various wavelet bases, such as Morlet, Mexican hat, Meyer, Daubechies, Symlets and Coiflets, Haar and Biorthogonal wavelets. It is vital to choose a suitable wavelet base and appropriate number of decomposition levels for the signal of interest using the wavelet transform. Different wavelet bases have their characteristics and are suitable to a specific application [64], for example, the Haar wavelet is simple and fast while resulting in memory efficiency, and it can separate signal and noise without significant loss of the signal information. Meyer wavelet is endowed with better localization characteristics, while biorthogonal wavelet is useful for signal reconstruction because it has linear phase filter banks with symmetric property. Daubechies wavelet possesses symmetry with the energy spectrum mainly distributed around low frequencies, which is found more suitable for the detection of R-peak. In practice, the appropriate wavelet function should be selected according to the parameters such as supporting length, symmetry, vanishing moment, regularity and similarity [18,65]. A reasonable selection of wavelet bases should pay proper consideration to these properties for a specific application.

In wavelet family, discrete wavelet transform (DWT) is easy to work on a computer, and the calculation efficiency is relatively higher compared with other types of wavelets. DWT is also the most widely used in the analysis of ECG [66,67,68,69,70]. Jayachandran et al. took advantage of multiresolution properties of wavelet transformation to identify subtle changes in the ECG signal for detection of myocardial infarction (MI) [71]. They used DWT to decompose ECG into various resolution levels. By comparing energy–entropy characteristics of ECG signal from 2282 normal and 718 MI in the wavelet domain, the proposed method discriminated the normal and MI ECG beat with more than 95% accuracy. Hong et al. applied discrete wavelet packet transform (DWPT) to perform feature extraction on 12-lead ECG with high-dimensionality and multiple signals [72]. It was found that wavelet tensor-based multilinear principal component analysis (MPCA) could extract features more efficiently than vector-based PCA. There are still many wavelet analysis methods that have been proved to be very effective in ECG analysis. Mohit Kumar et al. applied flexible analytic wavelet transform (FAWT) technique to feature extraction, and computed cross information potential (CIP) parameter from the real values of the coefficients of FAWT to capture the hidden information from ECG beats [73]. High classification accuracy was usually achieved by optimizing decomposition level. However, the accuracy of DWT method often depends on the assumption that the shape of the basis function is similar to that of the ECG signals [10]. It is difficult to predict the shape of the basis function, especially for non-stationary ECG signals.

#### 3.1.4. Statistical and Morphological Features

Statistical features, such as mean value, variance, deviation, Shannon entropy, have also been proved to be effective in ECG analysis [74,75,76,77,78]. The statistical features are low complexity and independent of fiducial points of the ECG signals. Thus, they are usually unaffected by fake fiducial points of the ECG signal. Besides, the statistical features are also used in detection of noise artifacts to pick up signals for improving accuracy. Lee et al. developed a real-time method for detection of motion and noise (MN) artifacts [79]. They separated high-frequency components using first-order intrinsic mode function(F-IMF). Then, the Shannon entropy, mean, and variance calculated from the F-IMF time series were selected as features of MN artifacts to detect the presence of MN artifacts with sensitivity of 96.63%. Morphological features of ECG signals refer to the indicators obtained by the ECG after mathematical morphological processing, which are usually achieved by morphological filtering [55,80,81,82,83,84,85,86,87]. As a kind of nonlinear transformation, the morphological filter can locally modify geometric characteristics of a signal. One of the most important concepts in morphological filtering is a structural element, called probe. The relationship between the parts of the signal is checked by moving the probe. Compared with the time-frequency transform method, appropriate structural elements can preserve the signal well. However, most of the statistical features and morphological features are not comprehensive and prominent, and most of the studies do not use these features alone, but often use them in combination with other kinds of features, or use statistical methods to further extract the existing features [77,88,89,90]. Raj et al. combined dictionary decomposition method with statistical characteristics, and proposed an overcomplete Gabor dictionary-based statistical feature extraction method [91]. ECG signals were decomposed into elementary waves by the overcomplete Gabor dictionary. Four statistical features, including time delay, frequency, width parameter, and square of expansion coefficient were extracted from the elementary waves. These features were fed to classifier models, and optimized least-square twin SVM, achieving a high classification accuracy of 99.11%. This method combining dictionary decomposition and statistical characteristics, significantly improved the ECG detection speed, reduced computation, and improved accuracy. Compared with first-order and second-order statistics (e.g., mean and variance), higher-order statistics (HOS) have attracted extensive attention in the field of ECG analysis. Higher-order statistics are proved to be suitable for estimation of skewness and kurtosis, and are blind to additive Gaussian noise. Marinho et al. used statistical methods including HOS and structural-co-occurrence matrix for features extraction, and used classifiers (support vector machine, multi-layer perceptron, bayesian, and optimum-path forest) to discriminate arrhythmias [92]. The feature set based on HOS obtained the highest accuracy compared with other features. The combination of HOS and Bayesian classifier achieved the highest accuracy of 94.3%. The results demonstrated that it is clinically reliable to use HOS for describing types of arrhythmia.

### 3.2. Dimensionality Reduction

High dimensionality of the feature space can provide much more detailed information of ECG signal. However, the higher the number of data, the more computational cost. Some of the features may be correlated, resulting in a large number of irrelevant variables, which will significantly affect computational efficiency due to the large redundant data [93]. Hence, it is vital to remove some correlated features while improving the accuracy and efficiency of classification. Dimensionality reduction makes data analysis much simple and fast, thus improving the performance of clustering algorithms with reduced features. Usually, the dimensionality reduction is accomplished based on either feature selection or feature extraction.

#### 3.2.1. Feature selection

**Figure 3:**
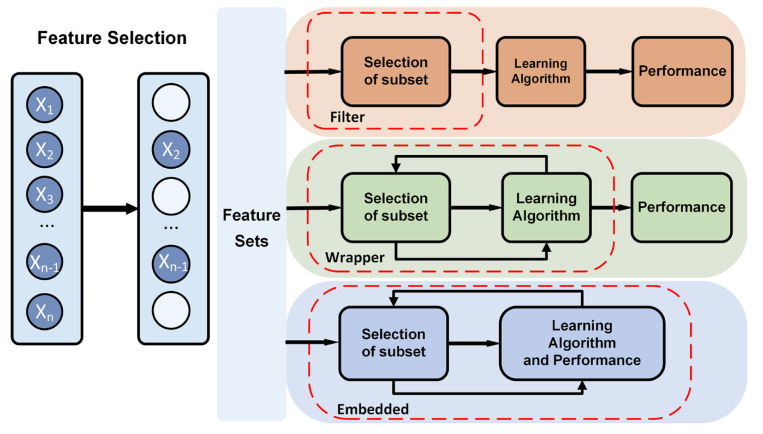
Feature selection methods, such as filter, wrapper, and embedded method.

Feature selection tries to select a subset of the original feature set, which efficiently describes the input data and makes weak correlation among these features [94]. Therefore, the focus of feature selection is to find appropriate standards or algorithms to evaluate the contribution of features to the results, thus reducing the dimension of features for improving the model generalization ability and reducing overfitting. Feature selection usually involves three ways, including filters, wrappers and embedded (Figure 3).

Filter-based feature selection applies a selected metric to find irrelevant attributes and filters out the redundant data [95]. The selection process is independent of the training process. Filter-based methods rank the features as a pre-processing step before the learning algorithm, and select those features with high ranking scores. The score is computed by measuring the variance between the expected value of the information and the observed value. The evaluation metric of filter usually is used to analyze the internal features of the feature subset, which includes correlation, distance, information gain, and so on. In practice, filter-based feature selection can be initially screened by expert knowledge, then filtered by filtering methods. The characteristics that have been proven to be relevant to a particular disease or physiological response are often directly selected in feature selection. Besides, it is a common method to calculate a score for each feature column. Columns with poor feature selection scores are ignored. Filter-based feature selection provides a variety of performance criteria for assessing the information value, such as correlation coefficient, mutual information, Kendall correlation, Spearman correlation, Chi Squared, Fisher score, Laplacian score, Trace Ratio criterion, among which, Fisher score is widely used metrics for supervised feature selection. Fahim Sufi et al. ranked feature subsets according to a correlation-based heuristic evaluation function [96]. The algorithm selected features by calculating mean feature-class correlation and the average feature-feature intercorrelation. The two criteria ensure that irrelevant features and redundant features are removed from the attributes set, because they are not correlated with the class or other features. Some researchers evaluated each feature from the primary feature sets by Fisher score [97,98]. The fisher score selected each ECG feature independently according to their scores under the Fisher criterion, resulting in suboptimal subset of attributes. It is often used to select feature sets with lower dimension. The Filter method uses statistical indicators to score and filter each feature, focusing on the characteristics of the data itself. The advantage of the Filter method is that the calculation is fast and does not depend on a specific model. However, the final accuracy of the classification may be not high because the selected features are not customized for the specific model

Wrapper-based feature selection utilizes a predefined classifier to evaluate the feature set. This method scores the features using the learning algorithm that will ultimately be employed in classification. The feature selection process is integrated with training process, and the prediction ability of the model is used as the selection criterion to evaluate the feature subset, such as classification accuracy, complexity penalty factor. Forward and backward selection algorithm in multiple linear regression is a simple implementation of wrapper. Sequential floating forward search (SFFS) algorithm utilizes sequential forward selection (SFS) and sequential backward selection (SBS) in sequence to obtain the best ECG feature set. Llamedo and Martinez obtained an optimal ECG feature set containing eight features by SFFS [99]. The SFFS method is suitable for small-and medium-scale data [100]. KNN [101] and SVM [102] can be used as evaluation functions of the wrapper. Compared with the filter, the wrapper has better performance in generating high-quality subsets, but the data processing is computationally expensive since the learner needs to be trained many times during the feature selection process. Unlike filter selection, which does not consider subsequent classification algorithms, wrapped selection directly takes the performance of the final classification algorithms as the evaluation standard of the feature subset. In other words, wrapped feature selection is to select the most favorable feature subset for a given learning algorithm. However, the performance of the subset of features is affected by the particular learning algorithm. The stability and adaptability of the feature subset are poor because each additional feature must be constructed feature subset for evaluation. Wrapper-based feature selection has high time complexity and is not suitable for high dimensional data set.

Embedded feature selection is built into the construction of the machine learning algorithm. It provides a trade-off solution between filter method and wrapper method, which can solve the high redundancy of the filter algorithm and the computational complexity of the wrapper algorithm. The embedded feature selection is automatically performed during the learner training process [96]. Compared to the other two methods, the searching and selection process of features’ subset is built into classifier construction. Regularization and tree-based methods are widely used in embedded methods. The regularization models in form of ℓ_2, 1_-norm regularized regression models, such as Lasso, sparse linear discriminant analysis, and regularized support vector machine, are widely used in embedded methods [103]. Regularization is to impose additional constraints or penalties on the loss function when training a neural network, which can reduce the complexity and instability of the model in the learning process, thus avoiding overfitting and improving generalization ability. Decision tree is a classic embedded feature selection method, such as ID3, C4.5, CART algorithm. Features with good ability of classification are selected in the nodes of the tree, and then the selected feature subsets are used to perform the learning tasks. Feature subsets are selected during the process of decision tree generation. The random forest has the advantages of high accuracy, good robustness and easy to use, which makes it one of the most popular machine learning algorithms. The random forest provides two methods of feature selection, including mean decrease impurity and mean decrease accuracy. Tree-based prediction models can be used to calculate the importance of features, and thus to remove irrelevant features. Embedded feature selection can be applied to high dimensional data sets, but the design of the embedded method is tightly coupled with a specific learning algorithm, which in turn limits its application to other learning algorithms.

#### 3.2.2. Feature Extraction

The disadvantage of feature selection is that unselected features are simply moved out, which will reduce the accuracy and efficiency of learning algorithms. Feature extraction considers all features and maps the useful information into a low-dimensional feature space, which is more commonly used in the selection of feature sets with insufficient prior knowledge and high-dimensionality. By choosing an appropriate dimension reduction method, the invalid information of the original feature set can be removed and the effective information of the original feature set is retained to the greatest extent. Typical dimensionality reduction methods include principal component analysis (PCA), linear discriminant analysis (LDA), independent component analysis (ICA), and generalized discriminant analysis (GDA) [104].

The PCA method is a linear dimensionality reduction method that maps the original features into a low-dimensional space while retaining the variance. PCA is the most widely used form of dimensionality reduction, which preserves the maximum amount of variance of the original data. The choice of optimal number of principal components is one of the major challenges for providing meaningful interpretation of time series. He and Tan developed entropy-based adaptive dimensionality reduction and clustering methods for automatic pattern recognition of ECG signals [105]. A novel entropy-based principal component analysis (EPCA) was developed to automatically select the optimal number of principal components for dimensionality reduction of ECG signals. Then, a novel fuzzy entropy c-means clustering algorithm (FECM) was utilized to identify the best number of clusters for a specific subject. The results on ECG signals verify that the performance of EPCA is superior to PCA based on cumulative percentage and screen graph. The clustering accuracy of FECM performs superiorly to Ng–Jordan–Weiss (NJW) method, hierarchical agglomerative clustering (HAC) and K-means with the known cluster number. LDA also known as Fisher’s discriminant analysis, is a dimensionality reduction algorithm. Unlike PCA, LDA is a supervised algorithm that maximizes separation between multiple classes, while PCA is an unsupervised algorithm that focusses on maximizing variance in a dataset. PDA shows good performance in pattern recognition. Varatharajan et al. applied LDA to reduce the features of the ECG signal [106]. The effectiveness of LDA with an enhanced kernel-based SVM method was proved by calculation of sensitivity, specificity and mean square error. LDA is applied to DWT sub-bands for dimensionality reduction by Martis et al. [68]. By using 12 linear discriminant features as input, both neural network and SVM achieved average classification accuracy of more than 97%. ICA is a linear dimension reduction method, which transforms the features into columns of mutually independent components [107,108]. Independent components can be picked up from the mixed signals by ICA. Martis et al. compared the performance of various dimensionality reduction techniques for arrhythmia classification, including PCA, LDA and ICA, based on DWT features [69]. ICA coupled with neural network yielded the highest average sensitivity, specificity, and accuracy of 99.97%, 99.83% and 99.28%, respectively. Experimental results showed that the ICA on DWT coefficients was more robust and yielded good classification accuracy. The linear feature extraction methods are relatively simple, however, the feature extraction model will be wrong by projecting data onto a linear subspace when the dataset has non-linear connections.

**Figure 4:**
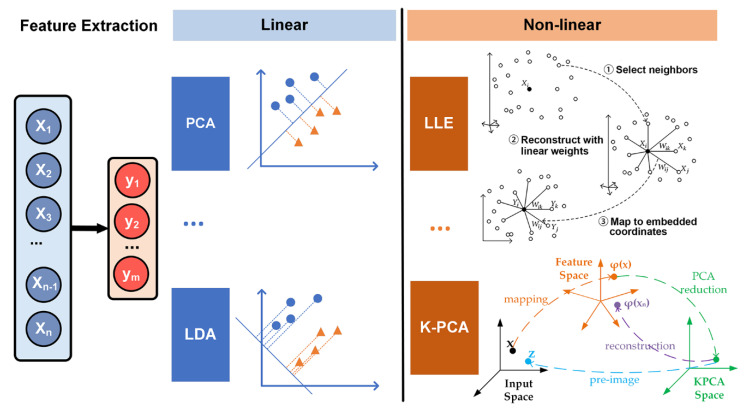
Different feature extraction methods used in ECG analysis.

In clinical practice, all the changes of ECG parameters are detected by visual evaluation and manual interpretation to detect the presence of cardiovascular disease. However, due to the nonstationary and nonlinear nature of ECG signals, cardiovascular disease indicators may appear randomly on the time scale. Non-linear features of ECG, such as energy (Ee), entropy (Ez), fractal dimension (F_D_), and relative wavelet (RWz) can be extracted to show some diagnostic details that can’t be simply detected by visual evaluation [109]. Nonlinear methods can perform better in complex nonlinear relationships among the features [110]. Locally linear embedding (LLE) is an unsupervised nonlinear dimensionality reduction method, which tries to preserve the data structure by a non-linear method according to local features of the dataset (Figure 4). 

It can perform much faster than the other approaches. However, LLE is extremely susceptive to noises, and LLE will not preserve well the local geometry of the data sets in the embedding space if there are outliers in the data [111]. Kernel-based method has become one of the most popular approaches to extract the complicated nonlinear information embedded on an ECG dataset. Kernel LLE (KLLE) can reconstruct nonlinear data as a linear combination of its neighbors. Li et al. mapped 12-dimensional features of ECG segments of single beat type into 7-dimensional embedding space described by two coordinates of kernel LLE [112]. The results showed that the true/false ratio for the proposed method outperformed other selected methods. The KLLE space shows better performance in loss of diagnostic information in ECG signals. Kernel principal component analysis (KPCA) is a popular nonlinear generalization of PCA, which is suitable for processing linearly nonseparable data sets. The basic idea of KPCA is to map the original data into a high dimensional space via a kernel function (Φ), and then to apply the standard PCA algorithm to it (Figure 4) [113], which can extract a more complete nonlinear representation of the principal components. However, KPCA takes more time than PCA.

## 4. Classification

Classification model is responsible for making judgments based on a series of input data, thus ultimately achieving a goal of disease diagnosis. The early classifiers consisting of a series of thresholds and judgment sentences can simulate the human logic to perform some simple tasks [114]. The early models are highly interpretable, but they cannot effectively analyze complex tasks. Machine learning models currently become mature and can maintain more than 90% accuracy in arrhythmia detection. However, in the analysis process, feature engineering is still labor-intensive, and needs excessive processing steps which greatly weaken the effective information of the data. Deep learning models are powerful analytical models that have emerged in recent years. Although this type of model is computationally expensive, it is powerful and can greatly reduce the use of artificial features. The End-to-End analysis based on deep learning models can directly process raw data without hand-crafted techniques, which provides the possibility for fully automatic analysis of ECG, especially in the classification of complex problems [14].

### 4.1. Machine Learning Classifier

Machine learning is the use of algorithms to parse data, and then make decisions and predictions about events in the real world. Commonly used machine learning algorithms include K nearest neighbor (KNN), decision tree, random forest, logistic regression, support vector machine (SVM), naive Bayes, K mean algorithm, adaboost algorithm, neural network, Markov, and so on. KNN is a relatively simple clustering method that can divide points into multiple categories based on distance. However, its calculation complexity is high for high-dimensional data, and it is difficult to overcome the impact of data imbalance. Decision tree and random forest continuously split the data according to the target function to find the best segmentation which minimizes the overlap of classes. Decision Trees are highly interpretable, but its accuracy will be seriously affected when the data is unbalanced. SVM as a popular approach has been used in pattern classification with high feature dimension and small training set, achieving good generalization ability [115]. It shows many unique advantages in solving small samples, nonlinear, and high-dimensional pattern recognition [116,117,118]. Polat and Güneş applied principal component analysis (PCA) to reduce 279-dimensional features to 15 dimensions, and used least square support vector machine (LS-SVM) for detection of ECG arrhythmias [119]. The obtained classification accuracy is 100% with 70–30% of training-test dataset or 80–20% of training-test dataset. Performance of the model can be improved by multiple machine learning classifiers for decision optimization. Osowski et al. proposed a highly reliable expert system based on two SVMs for heartbeat recognition. The system applied higher order statistics (HOS) and Hermite characterization of QRS complex for generation of features, and the two subsets of features were fed into SVM classifier, forming two neural classifiers. Least mean square method was applied to optimize the weights of the weighted voting integrating scheme for the combination of the two classifiers. The error rate of 4.09% after fusion was significantly lower than that of the two classifiers, which were 5.43% and 6.28% respectively [120]. Ye et al. proposed a two-lead fusion method based on probability estimation for automatic heartbeat classification [104]. The procedure was independently applied to the data from two ECG leads and the two decisions were fused for the final classification decision. The method achieved a high accuracy rate of 99.3% based on MIT-BIH, and effectively reduced the false detection rate.

Artificial neural network (ANN) as a kind of computational algorithm inspired by networks of biological neurons has been developed to solve prediction problems in ECG arrhythmias classification, due to its strong robustness and fault tolerance to noise. The neural networks are suitable for approximating complex nonlinear relations. Pinho André et al. simultaneously tested ANN and SVM using the different sets of features for sleep apnea detection. The results showed that the performance of ANN was better than SVM [11]. Multi-layered perceptron (MLP) recognizes and classifies ECG signals more accurately than other models of ANN. Moavenian et al. compared SVM and multi-layered perceptron (MLP) with a backward neural network in the classification of arrhythmia. Although SVM performed better in speed, its accuracy and specificity were slightly lower than MLP [121]. The learning ability of the ANN increases with the number of layers. However, gradient dissipation and computing ability of machines limit its performance. With the solution of gradient dissipation and the enhancement of computer computing power, the advantages of neural networks have gradually expanded. A deep neural network (DNN) is a particular artificial neural network with more than one layer of hidden units between input layer and output layer. Compared with machine learning methods such as SVM, DNNs are better at handling large-scale, high-dimensional data, and are more robust. With different network structures, the neural network method can analyze data with lower labor costs. Sannino et al. proposed a DNN with 7 hidden layers for the classification of abnormal ECG beats. The accuracy of the model for arrhythmia detection reached 99.09%, which was higher than eleven machine learning classifiers and was also competitive in terms of sensitivity and specificity, 98.55% and 99.52%, respectively [122]. Currently, there is no standard methodology for the construction of an optimal neural network with the correct number of layers and neurons for each layer. Most of the DNNs are built by experience. Hasan et al. used EMD to decompose ECG signals in time domain into inherent modal signals (IMFs), and employed DNN as a classifier. The classification accuracy on three datasets of MIT-BIH, St-Petersburg and PTB is 97.70%, 99.71% and 98.24%, respectively [35]. Nevertheless, the limitation of these methods that require manual feature selection and prior domain knowledge. Therefore, data scientists still need to carefully create features to achieve good performance.

### 4.2. End-To-End Model

The existence of various noises, the deviation of prior knowledge, feature extraction methods, and the choice of dimensionality reduction methods affect the quality of features. Therefore, it is necessary to optimize the parameters of each step to obtain excellent performance. Currently, although many models have been developed for a specific task with good performance, the generalization performance of computational algorithms is still not satisfactory, and most of the algorithms need to be redesigned for a specific task, devices, and even application scenarios. Steady increase in computing power and availability of large volumes of data has driven the development of machine learning profoundly. End-to-end models based on DNNs are currently the most important artificial intelligence design solutions. Raw data is directly fed into DNN model without creating any specific features to realize end-to-end analysis. With multiple layers of neurons, DNN automatically generates appropriate features and achieves good performance with a large amount of data. It shifts the burden of feature engineering to the learning body itself and provides opportunities for the accuracy and scalability of the ECG analysis. DNN has also evolved into many different network topologies, such as convolutional neural network (CNN), recursive neural network (RNN), and long-term short-term memory (LSTM).

**Figure 5:**
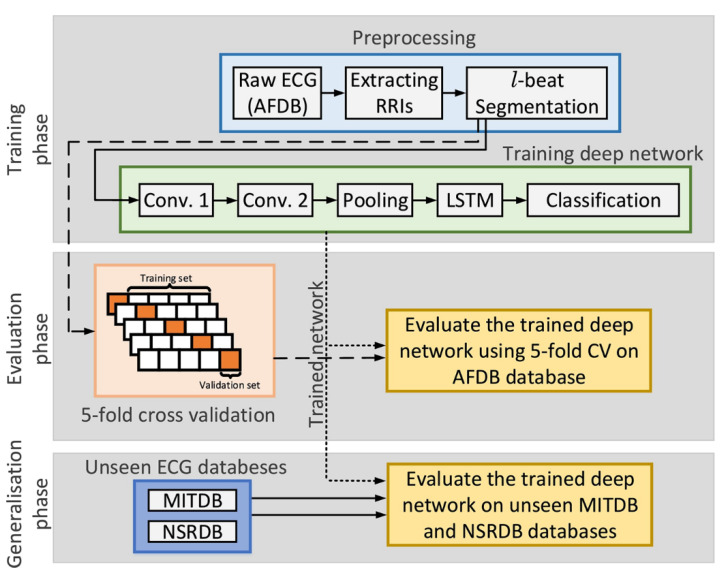
Flowchart of the Convolutional- and Recurrent-Neural Networks (reproduced with permission from the authors of [129]). The model consists of a training phase for estimation of the optimal parameters of model, an evaluation phase for validating performance measures and a generalization phase to report performance on previously unseen data sets.

The end-to-end model provides the possibility for complete intelligence of ECG analysis, which inherently fuses feature extraction and classification into the learning body and outputs decision-making. Based on 91,432 single-lead ECGs from 53,549 patients, Hannun’s team developed a DNN to distinguish 12 different heart rhythms [14]. They compared the classification sensitivity of the DNN model with the average specificity achieved by cardiologists. The results showed that the area under the receiver operating characteristic curve (ROC) of the model was 0.97. The F1 value of the model was 0.837, which was higher than the cardiologist’s average of 0.780. These findings demonstrated that the proposed end-to-end deep learning approach could classify a broad range of distinct arrhythmias with high diagnostic performance similar to that of cardiologists [123]. The improvement may be attributed to the feature learning capability of DNN, which can realize the function of feature extraction and classification. CNNs consisting of hierarchical neural networks are used for both feature extraction and classification. Its convolutional layers alternate with subsampling layers, which can learn to extract patient-specific features. The fully connected layers following the convolutional layers are identical to MLP. These layers perform the classification task to produce the final decision. Recent researches focus on ECG analysis with End-to-End approach were listed in Table 2. Kiranyaz et al. proposed an adaptive 1D CNN with three CNN layers and two MLP layers for real-time ECG classification of an individual patient [124], which avoids the need for any manual feature extraction and postprocessing, therefore, it shows great potential for a real-time implementation for heart monitoring because of its relative computational complexity. Zhai et al. proposed a 2D CNN network for arrhythmia classification [125]. Compared with the 1D-CNN proposed by Kiranyaz et al., this model improves the sensitivity of supraventricular ectopic beats (SVEB) by more than 12.2%. The matrix captures the morphology of a single heartbeat and the time relationship in the adjacent heartbeats by converting the three adjacent heartbeats in the ECG into a 2D coupling matrix. Hence, compared with 1D CNN, this method can more effectively investigate the relationship between various ECG components in adjacent heartbeats, thereby improving the performance of the classifier. In addition to CNN, recurrent neural network (RNN) can take full use of time-domain information, so it is widely used in natural language learning and signal analysis. Long short-term memory (LSTM) as a popular type of RNN that retains time domain information, which is prominent in the processing of sequential data. Tan et al. applied CNN to extract ECG features, and LSTM as a classifier to automatically diagnose CVD [126]. By using three-layer LTSM instead of CNN as the output layer, the model has better diagnostic performance than the 11-layer CNN model proposed by Acharya et al. [127]. Yildirim proposed a model with good effectivity and low computational cost to recognize ECG arrhythmia signals [128]. A deep convolutional auto-encoder (CAE) based compression model combined with LSTM networks to recognize ECG beats, which significantly reduced the training from 4.5 h to 0.6 h. Compared with the classic ML technology, reinforcement learning provides a faster learning mechanism and is more adaptable to changes in the environment. Andersen et al. provided a robust real-time End-to-end model including CNN and LSTM for automatic detection of atrial fibrillation in long-time ECG recordings [129]. The model consisted of a multi-layer deep learning network featuring convolutional and recurrent layers (LTSM, Figure 5), which was computationally efficient. It could analyze 24-h ECG recordings in 0.92 s. The model achieved sensitivity of 98.98% and specificity of 96.95%. The model trained by the MIT-BIH AF database performed AF detection on MIT-BIH arrhythmia database and MIT-BIH NSR database for evaluating the robustness of the model. Experimental results showed that the accuracy of the model hardly decreased in sensitivity, and the accuracy decreases only 10% in specificity, indicating that the model has good robustness on different data sets. The robustness of the model reflects the anti-interference ability to data disturbance and noise. In feature engineering, the development of features usually matches with the data sets and the specific tasks. Improper processing of noise and other interference will seriously affect the quality of the features and the accuracy of the model. The end-to-end neural network takes raw data as input, which is much more robust than feature engineering. Acharya et al. proposed a 11-layer deep CNN for diagnosis of myocardial infarction based on ECG data with and without noise, and achieved accuracy of 93.53% and 95.22%, respectively [130]. The experiment showed that the presence or absence of noise hardly affected the accuracy of the end-to-end neural network, with a difference of less than 2%, which reflects the amazing robustness of the end-to-end neural network. Besides, the end-to-end model also solves some problems that traditional analysis methods are difficult to solve. Traditional analysis methods usually analyze data of a specified length, such as single-beat ECG data, which leads to the loss of time domain information and is not conducive to adjustment and modification. Compared with traditional analysis methods, the End-to-End model can input long-term data at a time, and the algorithm barely changes, which is beneficial to expansion and modification. Acharya et al. developed a CNN-based neural network for detecting arrhythmia. The network supported input of 2 s or 5 s of ECG data, achieving accuracy of 92.50% and 94.90%, respectively [131]. This showed that long-term ECG data can improve the effectiveness of the classifier to a certain extent. The end-to-end model can also be used to solve individual automatic optimization problems. In the method based on feature engineering, the automatic optimization of individuals is one of the most difficult tasks, because the distribution of training data and the strong offset between patients leads to poor performance of the model in practice [86,132,133]. 

Carrera et al. provided an end-to-end model that can automatically adjust each user’s parameters by using fine-tuning techniques in deep learning to make the model perform superior on individual patients [143]. Once a dedicated model is trained for a specific patient, it can be potentially used alone to quickly and accurately classify a long ECG data stream, which is greatly beneficial for wearable ECG monitoring. Attia et al. trained CNN networks based on ECG data for identifying patients with asymptomatic left ventricular dysfunction (ALVD). The network model not only screened out ALVD, but also found patients with a 4-fold risk of developing ALVD in the future [144], which provides users with a low-cost, non-invasive ALVD screening tool.

End-to-end neural networks also have some disadvantages. First, because there is no prior knowledge to support, end-to-end neural networks rely on high-quality data sets compared to classic machine learning methods. A larger and more comprehensive data set will help the neural network to fit well. Besides, neural networks with deep architectures, which involve a large number of parameters, easily overfit the training data. Hence, certain training skills are required in the training process. Third, the model becomes a black box, which reduces the interpretability of the neural network. The model design can only be modified by model structure and training methods to obtain better results. However, because of its powerful functions, end-to-end neural networks will still be one of the main research directions of ECG analysis.

## 5. Electrocardiogram (ECG) Databases

A wide variety of standard ECG databases are available online for assessment of computational algorithms in ECG signal analysis [2,3], such as Massachusetts Institute of Technology-Beth Israel Hospital (MIT-BIH) arrhythmia database, Physikalisch-Technische Bundesanstalt diagnostic ECG database (PTB), American Heart Association (AHA) database, St.-Petersburg Institute of Cardiological Technics Database (INCART), University of Toronto Database (UofTDB), Fantasia Database, and so on. MIT-BIH databases provided by MIT consists of several subsets, such as MIT-BIH ST Change database, MIT-BIH atrial fibrillation database, MIT-BIH arrhythmia, MIT-BIH long term database and MIT-BIH supraventricular arrhythmia. The MIT-BIH arrhythmia is well-known and most widely used by researchers. The database was taken from 47 subjects at a sampling rate of 360 samples per second with 11-bit resolution. Each segment is half an hour long. Table 3 summarizes the main public databases available for ECG analysis in detail. Different methods (e.g., sampling frequency, sample number, and leads) for collecting ECG data are utilized by these databases. However, the limited number of public ECG databases are still the main limitation in ECG analysis. Setting up standard and new publicly available databases that contain a large number of patients with labels acquired using the latest medical guidelines is urgent for the development of computational algorithms for ECG based diagnosis.

## 6. Applications

ECG records the electrical signals of myocardial cells in each cardiac cycle, which contains a large amount of information related to the heart. Hence, it is widely used in the field of medical healthcare, such as diagnosis of heart disease, prediction of cardiovascular disease, stress and sleep monitoring, and wearable device based online health monitoring.

### 6.1. Disease Diagnosis

Coronary heart disease is the leading cause of death. The world health organization classifies coronary heart disease into five categories, including asymptomatic myocardial ischemia, angina, myocardial infarction, ischemic heart failure, and sudden death. The main cause of coronary heart disease is coronary atherosclerosis, which is an inflammatory response to chronic multifactorial injury of the vessel walls, resulting in the formation of atherosclerotic plaques. These deposits cause the surface and lumen of the coronary arteries to narrow, and impede the flow of blood. Over time, these deposits can break up and then clot the blood, leading to a fatal heart attack [145]. Early detection and treatment of CVDs can save lives and may help to prevent it from further deterioration. Computer-aided techniques provide a faster and accurate tool for identification of CVD from ECG signals. Kumar et al. developed an accurate, fast, and automated method to diagnose CVD using ECG signals [73]. They found a significant difference in cross information potential (CIP) of detail coefficients between normal and coronary hearts after flexible analytic wavelet transform (FAWT) decomposition. They adopt least squares support vector machine (LS-SVM) with radial basis function (RBF) and morlet wavelet kernels to obtain CIP features, which were used to characterize coronary artery disease. The accuracy of the classification was 99.60%, demonstrating that the developed methodology could be used in mass cardiac screening and could aid cardiologists in performing diagnosis. Patidar et al. proposed application of higher-order statistics and spectra (HOS) for an automated classification of normal and CVD conditions using ECG signals [146]. The proposed method has achieved 98.17% accuracy by k-nearest neighbors (KNN) classifier using 13 bispectrum features. Similarly, 31 cumulant features were subjected to decision tree (DT) classifiers with 98.99% average accuracy. They further developed an integrated index named Coronary Artery Disease Index (CADI) for automated discrimination of normal and CVD ECG classes. However, the proposed methodology was only verified using resting ECG signals without exercise ECG. Patidar et al. proposed a novel CVD risk index for diagnosis of CVD using centered correntropy based feature set extracted by tunable-Q wavelet transform (TQWT) [147]. The nonlinear features were computed on decomposed detail sub-band derived from ECG signals using TQWT. The experimental results demonstrated that the CVD index was significantly different for normal and CVD heart rate signals. They obtained the average classification accuracy of 99.7%, sensitivity of 99.6%, specificity of 99.8%. However, the method needs to undergo rigorous clinical validation with huge data before the system will be installed in hospitals and polyclinics for screening of CVD patients accurately. Acharya et al. used the improved binary particle swarm optimization (IBPSO) feature selection method to select important features for classification diagnosis [148]. They used analysis of variance (ANOVA) and relief techniques to rank the features and selected the first 20 features. The accuracy, sensitivity and specificity of this model were 99.55%, 99.93%, and 99.24% respectively using decision tree, and it could correctly differentiate CVD, myocardial infarction (MI) and congestive heart failure (CHF) from normal heart condition.

Cardiac arrhythmias happen when the electrical impulses can’t coordinate heartbeats properly, resulting in the heart to beat too fast, too slow or irregularly. It’s also one of the leading causes of death from heart disease. ECG trace analysis is helpful for most of the heart diseases sufferers. Albuquerque et al. applied an optimum-path forest (OPF) classifier to ECG-based arrhythmia classification [149]. Compared with SVM and Bayesian classifier, OPF was less generalist. While the SVM classifier was the most accurate, OPF achieved the best trade-off between computational load and recognition rate, which is more appropriate for the classification of arrhythmias in ECG signals in terms of the computational time for both training and test phases. The recognition of cardiac arrhythmia in minimal time is important to prevent sudden and untimely deaths. Sangaiah et al. developed a model for the analysis of cardiac arrhythmia [150]. The whole process involved three phases for arrhythmia classification, including ECG signal enhancement, feature extraction based on a devoted wavelet design, hidden Markov model (HMM). The proposed model achieved an overall accuracy of 99.7% and a sensitivity of 99.7%. The authors further claimed that the users can monitor their health during daily activities by combining the cardiac arrhythmia identification model with internet of medical things. Compared with the analysis method based on feature engineering, neural network has the unique advantages of robustness and ease of use. Romdhane et al. proposed a deep CNN model using a novel focal loss function for ECG heartbeat classification, which can perform feature extraction automatically and jointly with the classification. The focal loss function can effectively handle the imbalanced class problem by focusing the loss on minority classes. The results revealed that the focal loss function improved the classification accuracy and the overall metrics with accuracy of 98.41%, overall F1-score of 98.38% and overall recall of 98.41% [151]. Acharya et al. employed CNN technique to automatically detect arrhythmias, which eliminates the tedious work of pretreatment and feature engineering [131]. This method greatly reduces the complexity of the algorithm and makes the ECG analysis simple and friendly for end-users. As a result, the End-to-End neural network models are promising to be used as assistive tools for doctors in the future, although it often requires large amounts of data and computing power.

In addition to the most common diseases mentioned above, ECG can also be used to detect other diseases, such as diabetes [152], cardiopulmonary disease [136], sickle cell disease and thalassemia [153]. In general, ECG-based computer-aided disease prediction provides early warning to potential patients and reduces mortality to some extent.

### 6.2. Prediction of Cardiovascular Disease

Due to the sudden onset of cardiovascular diseases, ECG-based disease prediction is essential for identifying cardiac problems and avoiding sudden cardiac death [154]. The electrical signals of the heart correlate to the physiological operation. The subtle changes in myocardial electricity are believed to be related to the abnormal relaxation of myocardium, which may cause cardiovascular diseases. These changes are so subtle that it’s hard to be found on ECG. Hence, many cardiovascular diseases lose the chance of early diagnosis. Advanced signal processing skills are needed to extract subtle changes as features for screening cardiovascular disease. Partho et al. extracted 370 relevant features of ECG signal using wavelet transform and other signal processing methods to predict the left ventricular diastolic dysfunction (LVDD) [155]. According to the literature, the prediction of coronary heart disease, arrhythmia, especially atrial fibrillation, is the main application of ECG disease prediction [156,157]. The early diagnosis and prediction of coronary heart disease can improve the survival rate of patients. According to the fact that people with similar heart conditions almost have similar ECG signals’ patterns. Vafaie et al. proposed a new classification method based on dynamical model of the ECG signal to predict arrhythmias [158]. The method estimated new parameters of ECG signals and compared the similarity with the parameters of the known ECG signals, and the results indicated that the classifier could segregate the ECGs with an accuracy of 93.34%. Besides, a genetic algorithm was also applied to improve the accuracy in prediction of arrhythmia, achieving an accuracy of up to 98.67%. For most patients and potential patients, the typical AF signal lasts for a short time and is not easy to detect. Long-term monitoring is expensive and time-consuming, meanwhile, long-term monitoring is inconvenient and unnecessary for undiagnosed patients. Attia et al. designed a neural network model, which can find the tiny features in ECG, to recognize undetected patients with AF using the standard 10 s ECG [6]. The model was tested on 1,000,000 ECG fragments of 210,414 patients with an accuracy of 79.2% in the test dataset, and the sensitivity and specificity were both over 79%. It has good performance and can be applied to the hospital equipment for screening and early warning. Advancement in cloud computing and biomedical equipment, which provides large data storage and abundant computing power, enables rapid and accurate analysis of heart-related diseases. Venkatesan et al. extracted the heart rate variability (HRV) parameters of ECG, which was applied to an adaptive neuro-fuzzy inference system (ANFIS) to identify the risk of coronary heart disease. The method was based on cloud computing and analyzed ECG data collected from a wearable device automatically with an accuracy of 98.75% on classifying normal and coronary heart disease risk subjects [159].

### 6.3. The Trend of Portability and the Eise of Photoplethysmography(PPG)

**Figure 6:**
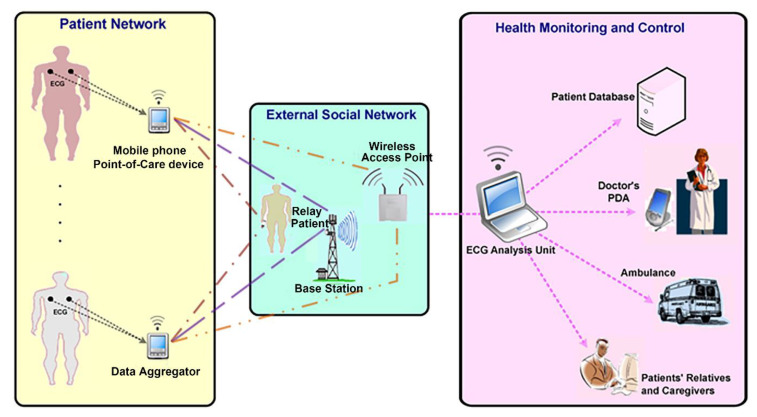
A wireless ECG monitoring system for E-health applications (reproduced with permission from the authors of [166]).

Wearable platform provides detailed longitudinal data for improving physical performance and positive habit formation [160]. ECG-based portable devices have become a research hotspot in recent years, which enable users to monitor their cardiovascular status at any time. Wearable devices provide continuous monitoring, health prediction and person-specific optimization in remote monitoring settings and smart homes, which makes up for the shortage of expensive equipment in hospitals. Early detection of CVDs requires long-term monitoring using ECG signals connected to a portable device that relies on wireless communication to external social networks (Figure 6). ECG signals collected from the wearable or portable device can potentially be added to databases via the existing internet. Clinicians perform diagnostic and screening procedures based on the computational analysis of ECG signals collected remotely for a person who needs continuous monitoring. It helps in identifying users who experience cardiac arrest and locating the position in real time. Many successful algorithms for cardiac function assessment based on ECG signals were studied and demonstrated their high accuracy. However, it is unclear which ECG analysis algorithm from the studies is suited for implementation on a portable device. Many problems still need to be solved before implementing these algorithms in wearable platforms, thus enabling online and long-term health monitoring. AF is the most common arrhythmia, with a prevalence of more than 3% in adults [161]. However, one third of them are subclinical [162]. Early detection of AF is an important objective for healthcare systems worldwide. According to the research of Aronsson [163], a large number of untreated AF can be detected by intermittent ECG recording using portable devices [164]. The relevant algorithms employed in a wearable device for long-time monitoring need to be optimized for daily activity scenarios. At present, ECG data sets are collected from patients in resting conditions in the hospital. However, since the morphology of the heartbeat varies with the heart rate, the algorithms trained in resting conditions need to be adapted to analyze ECG signals during daily activities. Carrera et al. proposed an online ECG monitoring solution. This method generates a user-specific sparse representation dictionary to represent the user’s normal heartbeats. The dictionary was then adjusted through a set of linear transformations independent of the individual user to track changes in heart rate. This method is of great practical significance because of its low computation and user-specific optimization [143]. Simplicity and efficiency are required in designing ECG-based models for processing long-term recordings and large databases [165]. A lot of works concerned online algorithms to detect CVDs automatically in real-time need to be done, thus providing real-time information to the hospital/doctor/patient when a critical heart condition occurs.

**Figure 7:**
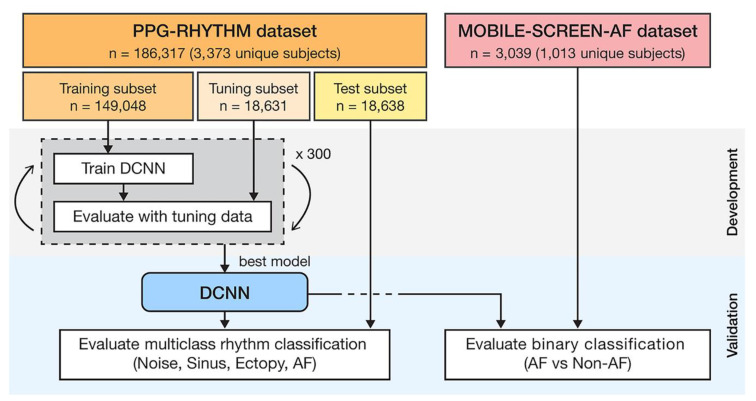
Workflow diagram showing the data sets used to develop and validate the DCNN in arrhythmia analysis (reproduced with permission from the authors of [137]).

Medical ECG devices usually use electrode patches to collect signals. However, these disposable electrode patches not only adhere directly to the skin, but also need to be connected to the device via a wire. Although the electrodes can provide excellent signal quality by employing the gel, adhesive, and metal stud, they are prone to skin irritation and discomfort during long-term use in daily life [167,168]. Photoplethysmography (PPG), as a noninvasive circulatory signal, is a highly popular alternative solution to ECG. The PPG technology is an optical technique that serves as an alternative to traditional ECG, which reflects ECG by measuring changes in the volume of blood vessels caused by beating heart [16,169]. PPG is a proven way to measure heart rate (HR), respiratory rate, tissue perfusion, and some vascular and heart diseases, which makes PPG technique very attractive in assessing vascular disease, especially for vascular aging, hypertension and atherosclerosis [170]. PPG also provides information about arterial stiffness and elasticity, and it is used to reconstruct ECG signal by exploiting the relation of these two types of cardiovascular measurement [171,172]. Although the HR measurements from ECG are more accurate than from PPG signals, PPG measurements are accurate enough for screening purposes, and are economically efficient, convenient, and easily integrated into wearable or portable devices for health monitoring. PPG is often employed in a non-invasive manner to make measurements at the skin surface. Its signal is superimposed on a series of low-frequency components, such as respiration, thermoregulation, the nature of skin tissues [173]. PPG-based cardiac analysis has been applied to various wearable devices [174]. Apple Watch uses PPG for data collection, which can continuously track users’ heart condition for 24 h [15]. Yang et al. proved that it is possible to achieve accurate detection of AF by using a small number of features extracted from PPG signals, enabling the use of affordable wearable devices [16]. PPG has been proven to be effective and practical [15,160,175,176,177,178,179]. The AF detection algorithm of Apple Watch has become the first consumer-oriented algorithm approved by the US food and drug administration (FDA) [180]. However, there is still much room for improvement in related technologies. For example, severe artifacts may occur during arm movement, which makes it possible to accurately classify related diseases only at rest. These artifacts will contaminate the PPG signal and limit the actual effectiveness of AF detection. Poh et al. trained a deep convolutional neural network (DCNN) for detecting atrial fibrillation using a large set of PPG signals [137] (Figure 7). This end-to-end model achieved better diagnostic performance than six other AF detectors based on features engineering. However, it is still needed to evaluate the DCNN model in a long-term ambulatory setting and verify its effectiveness for clinical decision-making and improving patient outcomes. At present, the application based on PPG has not reached the medical level and can only be used as a reliable reference. Currently, PPG used in wearable devices is often used to track daily health and exercise, such as sleep quality [179], exercise heart rate and stress detection [180]. Nevertheless, similar algorithms can still produce positive effects, which can remind users to seek medical advice in time [15], and show great potential in health monitoring.

## 7. Conclusions

In conclusion, we provide a summary of the latest computational diagnostic techniques based on ECG signals for estimating CVD conditions. The classic machine learning techniques play important roles in efficient and reliable monitoring of the ECG activity in hospital settings or at home by analyzing ECG recordings. The procedure of ECG signals analysis is discussed in several subsections, including data preprocessing, feature engineering, classification, and application. With the development of deep learning and other algorithms that need high computing power, the end-to-end models have been proposed for ECG analysis, which not only enable the analysis process no longer to require a feature extraction with hand-crafted techniques, but also have great advantages in accuracy and robustness. Compared to feature engineering based algorithms designed for a specific task, the end-to-end models have great potential to simultaneously perform multiple tasks and offer automatic optimization for a specific need. Notably, most successful models are trained by ECG signals in resting conditions collected by high-precision clinical equipment, which are supposed to be adjusted to learn from ECG signals recorded during everyday activities, challenges are still needed to be solved before employing these algorithms in wearable devices for online and long-term health monitoring. Identification and removal of motion artifacts in the dynamic ECG signals acquired by the wearable device will become a major problem. Their adaptability to real-time requirements and embed on wearable devices ensures an efficient and reliable monitoring of cardiovascular activity in hospital settings or at home. Computational diagnostic techniques for ECG signal analysis show great potential in helping health care professionals, thus improving care for CVD patients and helping to avoid potential catastrophic heart diseases.

## Figures and Tables

**Table 1:** ECG features and the normal values for a healthy adult.

Features	Description	Amplitude	Duration	Disease Diagnosis	References
R-R interval	The interval between two successive R-waves of the QRS complex ventricular rate		0.6–1.2 s	Paroxysmal atrial fibrillation Congestive heart failure	[45,46]
P wave	Atrial depolarization	0.25 mV	0.08–0.11 s	Atrial fibrillation Atrial hypertrophy	[47]
P-R interval	The time between the onset of atrial depolarization and the onset of ventricular depolarization		0.12–0.2 s	Stroke	[41]
QRS complex	Ventricular depolarization	1.60 mV for R peak	0.06–0.1 s	Ventricular enlargement Heart failure Tachycardia Acute Coronary Syndrome	[48,49,50]
ST-segment	The interval between ventricular depolarization and repolarization		0.05–0.155 s	Myocardial ischemia or infarction	[51]
T wave	Ventricular repolarization	0.1–0.8 mV	0.05–0.25 s	Myocardial infarction Pulmonary embolism	[46,52,53]
U wave	The last phase of ventricular repolarization	May not be observed because of its small size	Unknown	Unknown	[44]
QT interval	The time is taken for ventricular depolarisation and repolarisation		0.35–0.44 s	Hypokalemia ventricular arrhythmias	[54]

**Table 2:** ECG analysis with end-to-end approaches.

Tasks	Database	Model	Signal	Performance (%)	References
AF detection	MIT-BIH	CNN and RNN	250 samples	Acc = 97.10 Sen = 98.98 Spe = 96.95	[129]
Myocardial infarction detection	PTB	CNN	651 samples	Acc = 93.53 Sen = 93.71 Spe = 92.83	[130]
CVD detection	INCART	1D-CapsNet	514 samples	Acc = 99.44 Sen = 99.7 Spe = 98.1	[134]
MI classification	PTB	CNN	651 samples	Acc = 99.78 Sen:above 99 Spe:above 99	[135]
Arrhythmia detection	MIT-BIH	CNN	500 samples	Acc = 92.50 Sen = 98.09 Spe = 93.13	[131]
Classification of ECG signal	MIT-BIH	DNN	300 samples	Acc = 98.6 Sen = 92.4 Spe = 99.29	[132]
Classification of ECG signal	MIT-BIH	1D-CNN	128 samples	Acc = 99 Sen = 93.9 Spe = 98.9	[124]
Classification of ECG signal	A synthetic dataset by using an ECG simulator	Short-Time Fourier Transform and CNN	2426 samples	Acc = 99.2	[136]
AF detection	IEEE-TBME	CNN	512 samples	Acc = 96.1 Sen = 97.0 Spe = 100.0	[137]
Classification of ECG signal	TNMG	DNN	2800 samples	Spe:above 99	[138]
AF detection	iRhythm Technologies	DNN	256 samples	AUC:above 97 Sen:above 90 Spe:above 90	[14]
Classification of ECG signal	MIT-BIH	DNN	360 samples	AUC = 0.999 Acc = 98.97 Sen = 97.68 Spe = 99.89	[139]
AF detection	MIT-BIH	CNN	360 samples	Acc = 99.45 Sen = 99.29	[140]
MI detection	PTB	CNN	800 samples	Acc = 95.49 Sen = 94.85 Spe = 97.37	[141]
AF detection	MIT-BIH	CNN	3600 samples	Acc = 91.33 Sen = 83.91 Spe:above 99	[142]

Accuracy(Acc), Sensitivity(Sen), Specificity(Spe), Area Under Curve(AUC), MIT-BIH arrhythmia Database (MIT-BIH), Physikalisch-Technische Bundesanstalt diagnostic ECG database (PTB), St.-Petersburg Institute of Cardiological Technics database (INCART), IEEE-TBME PPG Respiratory Rate Benchmark data set (IEEE-TBME). Telehealth Network of Minas Gerais (TNMG).

**Table 3:** Brief description of ECG databases [2,3].

Database	Subjects	Records	Duration (min)	Frequency (Hz)	Leads	Resolution (bit)
MIT-BIH Arrhythmia	47	48	30	360	12	11
MIT-BIH AF	25	25	10 h	250	2	12
MIT-BIH ST Change	28	28	13–67	360	1–2	N/A
MIT-BIH Long Term	7	7	14–22h	128	2lead:12 1lead:10	2lead:6 1lead:3
MIT-BIH SUPRA	N/A	78	30	128	10	2
PTB	290	549	N/A	1k	12 + 3 Frank-lead	16
AHA	N/A	10	30	250	2	12
INCART	32	75	30	257	N/A	12
UofTDB	1020	1020+	2 to 5	200	1	12
Fantasia	40	40	120	250	N/A	N/A

## References

[1] Emelia J., Benjamin S.S.V., Clifton W., Callaway A.M.C., Alexander R., Chang S.C., Stephanie E., Chiuve M.C., Francesca N., Delling R.D. (2018). Heart Disease and Stroke Statistics—2018 Update: A Report from the American Heart Association. Circulation.

[2] Luz E.J.D.S., Schwartz W.R., Cámara-Chávez G., Menotti D. (2016). ECG-based heartbeat classification for arrhythmia detection: A survey. Comput. Meth. Prog. Biomed..

[3] Kaplan Berkaya S., Uysal A.K., Sora Gunal E., Ergin S., Gunal S., Gulmezoglu M.B. (2018). A survey on ECG analysis. Biomed. Signal Process..

[4] Besterman E., Creese R. (1979). Waller—pioneer of electrocardiography. Heart.

[5] Schijvenaars B.J.A., Kors J.A., van Herpen G., Kornreich F., van Bemmel J.H. (1997). Effect of electrode positioning on ECG interpretation by computer. J. Electrocardiol..

[6] Attia Z.I., Noseworthy P.A., Lopez-Jimenez F., Asirvatham S.J., Deshmukh A.J., Gersh B.J., Carter R.E., Yao X., Rabinstein A.A., Erickson B.J. (2019). An artificial intelligence-enabled ECG algorithm for the identification of patients with atrial fibrillation during sinus rhythm: A retrospective analysis of outcome prediction. Lancet.

[7] Robert C., Schlant M.C., Robert J., Adolph M., John P., DiMarco M.P., Leonard S., Dreifus M., Marvin I., Dunn M. (1992). Guidelines for electrocardiography: A report of the American College of Cardiology/American Heart Association Task Force on assessment of diagnostic and therapeutic cardiovascular procedures (Committee on Electrocardiography). J. Am. Coll. Cardiol..

[8] Schnabel R.B., Yin X., Gona P., Larson M.G., Beiser A.S., McManus D.D., Newton-Cheh C., Lubitz S.A., Magnani J.W., Ellinor P.T. (2015). 50 year trends in atrial fibrillation prevalence, incidence, risk factors, and mortality in the Framingham Heart Study: A cohort study. Lancet.

[9] Morrison W.G., Swann I.J. (1990). Electrocardiograph interpretation by junior doctors. Emerg. Med. J..

[10] Acharya U.R., Fujita H., Adam M., Lih O.S., Sudarshan V.K., Hong T.J., Koh J.E., Hagiwara Y., Chua C.K., Poo C.K. (2017). Automated characterization and classification of coronary artery disease and myocardial infarction by decomposition of ECG signals: A comparative study. Inf. Sci..

[11] Pinho A., Pombo N., Silva B.M.C., Bousson K., Garcia N. (2019). Towards an accurate sleep apnea detection based on ECG signal: The quintessential of a wise feature selection. Appl. Soft Comput..

[12] Ebrahimzadeh A., Shakiba B., Khazaee A. (2014). Detection of electrocardiogram signals using an efficient method. Appl. Soft Comput..

[13] Al Aref S.J., Anchouche K., Singh G., Slomka P.J., Kolli K.K., Kumar A., Pandey M., Maliakal G., van Rosendael A.R., Beecy A.N. (2019). Clinical applications of machine learning in cardiovascular disease and its relevance to cardiac imaging. Eur. Heart J..

[14] Hannun A.Y., Rajpurkar P., Haghpanahi M., Tison G.H., Bourn C., Turakhia M.P., Ng A.Y. (2019). Cardiologist-level arrhythmia detection and classification in ambulatory electrocardiograms using a deep neural network. Nat. Med..

[15] Isakadze N., Martin S.S. (2019). How useful is the smartwatch ECG?. Trends Cardiovas. Med..

[16] Yang C., Veiga C., Rodriguez-Andina J.J., Farina J., Iniguez A., Yin S. (2019). Using PPG Signals and Wearable Devices for Atrial Fibrillation Screening. IEEE Trans. Ind. Electron..

[17] Lyon A., Mincholé A., Martínez J.P., Laguna P., Rodriguez B. (2018). Computational techniques for ECG analysis and interpretation in light of their contribution to medical advances. J. R. Soc. Interface.

[18] Singh B.N., Tiwari A.K. (2006). Optimal selection of wavelet basis function applied to ECG signal denoising. Digit. Signal Process..

[19] Friesen G.M., Jannett T.C., Jadallah M.A., Yates S.L., Quint S.R., Nagle H.T. (1990). A comparison of the noise sensitivity of nine QRS detection algorithms. IEEE Trans. Biomed. Eng..

[20] Van Alste And T.S., Schilder J.A. (1985). Removal of base-line wander and power-line interference from the ECG by an efficient FIR filter with a reduced number of taps. IEEE Trans. Biomed. Eng..

[21] Oster J., Behar J., Sayadi O., Nemati S., Johnson A.E.W., Clifford G.D. (2015). Semisupervised ECG Ventricular Beat Classification with Novelty Detection Based on Switching Kalman Filters. IEEE Trans. Biomed. Eng..

[22] Appathurai A., Jerusalin Carol J., Raja C., Kumar S.N., Daniel A.V., Jasmine Gnana Malar A., Fred A.L., Krishnamoorthy S. (2019). A study on ECG signal characterization and practical implementation of some ECG characterization techniques. Measurement.

[23] Dalin Tang Z.T., Canton G., Hatsukami T.S., Dong L., Yuan X.H.A.C. (2009). Local critical stress correlates better than global maximum stress with plaque morphological features linked to atherosclerotic plaque vulnerability: An in vivo multi-patient study. Biomed. Eng. Online.

[24] Frolich L., Dowding I. (2018). Removal of muscular artifacts in EEG signals: A comparison of linear decomposition methods. Brain Inf..

[25] Blanco-Velasco M., Weng B., Barner K.E. (2008). ECG signal denoising and baseline wander correction based on the empirical mode decomposition. Comput. Biol. Med..

[26] Alfaouri M., Daqrouq K. (2008). ECG Signal Denoising By Wavelet Transform Thresholding. Am. J. Appl. Sci..

[27] Xu Y., Luo M., Li T., Song G. (2017). ECG Signal De-noising and Baseline Wander Correction Based on CEEMDAN and Wavelet Threshold. Sensors.

[28] Han G., Xu Z. (2016). Electrocardiogram signal denoising based on a new improved wavelet thresholding. Rev. Sci. Instrum..

[29] Üstündağ M., Gökbulut M., Şengür A., Ata F. (2012). Denoising of weak ECG signals by using wavelet analysis and fuzzy thresholding. Netw. Modeling Anal. Health Inform. Bioinform..

[30] Singh P., Pradhan G., Shahnawazuddin S. (2017). Denoising of ECG signal by non-local estimation of approximation coefficients in DWT. Biocybern. Biomed. Eng..

[31] Al-Betar M.A. (2017). β-Hill climbing: An exploratory local search. Neural Comput. Appl..

[32] Alyasseri Z.A.A., Khader A.T., Al-Betar M.A., Awadallah M.A. (2018). Hybridizing β-hill climbing with wavelet transform for denoising ECG signals. Inf. Sci..

[33] Huang N.E., Shen Z., Long S.R., Wu M.C., Shih H.H., Zheng Q., Yen N., Tung C.C., Liu H.H. (1998). The empirical mode decomposition and the Hilbert spectrum for nonlinear and non-stationary time series analysis. Proc. R. Soc. Lond. Ser. A Math. Phys. Eng. Sci..

[34] Satija U., Ramkumar B., Manikandan M.S. (2018). Automated ECG Noise Detection and Classification System for Unsupervised Healthcare Monitoring. IEEE J. Biomed. Health.

[35] Hasan N.I., Bhattacharjee A. (2019). Deep Learning Approach to Cardiovascular Disease Classification Employing Modified ECG Signal from Empirical Mode Decomposition. Biomed. Signal Process..

[36] Wu Z., Huang N.E. (2009). Ensemble empirical mode decomposition: A noise-assisted data analysis method. Adv. Adapt. Data. Anal..

[37] Chang K. (2010). Arrhythmia ECG Noise Reduction by Ensemble Empirical Mode Decomposition. Sensors.

[38] Kopsinis Y., McLaughlin S. (2009). Development of EMD-Based Denoising Methods Inspired by Wavelet Thresholding. IEEE Trans. Signal Process..

[39] Arumugam R., Jebaraj J. (2016). Ensemble empirical mode decomposition-based optimised power line interference removal algorithm for electrocardiogram signal. IET Signal Process..

[40] Liu S., Hsieh C., Chen W., Tan T. (2019). ECG Noise Cancellation Based on Grey Spectral Noise Estimation. Sensors.

[41] Montalvo M., Tadi P., Merkler A., Gialdini G., Martin-Schild S., Navalkele D., Samai A., Nouh A., Hussain M., Goldblatt S. (2017). PR Interval Prolongation and Cryptogenic Stroke: A Multicenter Retrospective Study. J. Stroke Cerebrovasc. Dis..

[42] Merone M., Soda P., Sansone M., Sansone C. (2017). ECG databases for biometric systems: A systematic review. Expert Syst. Appl..

[43] Marcinkevics R.O., Neill J., Law H., Pervolaraki E., Hogarth A., Russell C., Stegemann B., Holden A.V., Tayebjee M.H. (2018). Multichannel electrocardiogram diagnostics for the diagnosis of arrhythmogenic right ventricular dysplasia. EP Eur..

[44] Pérez-Riera A., Ferreira C., Filho C., Ferreira M., Meneghini A., Uchida A., Schapachnik E., Dubner S., Zhang L. (2008). The enigmatic sixth wave of the electrocardiogram: The U wave. Cardiol. J..

[45] Adami A., Gentile C., Hepp T., Molon G., Gigli G.L., Valente M., Thijs V. (2019). Electrocardiographic RR Interval Dynamic Analysis to Identify Acute Stroke Patients at High Risk for Atrial Fibrillation Episodes during Stroke Unit Admission. Transl. Stroke Res..

[46] Liu C., Gao R. (2017). Multiscale Entropy Analysis of the Differential RR Interval Time Series Signal and Its Application in Detecting Congestive Heart Failure. Entropy.

[47] Pürerfellner H., Pokushalov E., Sarkar S., Koehler J., Zhou R., Urban L., Hindricks G. (2014). P-wave evidence as a method for improving algorithm to detect atrial fibrillation in insertable cardiac monitors. Heart Rhythm.

[48] Bax J.J., Delgado V., Sogaard P., Singh J.P., Abraham W.T., Borer J.S., Dickstein K., Gras D., Brugada J., Robertson M. (2016). Prognostic implications of left ventricular global longitudinal strain in heart failure patients with narrow QRS complex treated with cardiac resynchronization therapy: A subanalysis of the randomized EchoCRT trial. Eur. Heart J..

[49] Brady W.J., Mattu A., Tabas J., Ferguson J.D. (2017). The differential diagnosis of wide QRS complex tachycardia. Am. J. Emerg. Med..

[50] Ibrahim Radwan H., Saad Mansour K., Mustafa Al-Daydamony M., Saed Mohammed R. (2019). Fragmented QRS Complex as a Predictor of High Risk in Acute Coronary Syndrome. Cardiol. Cardiovasc. Res..

[51] Hausenloy D.J., Botker H.E., Engstrom T., Erlinge D., Heusch G., Ibanez B., Kloner R.A., Ovize M., Yellon D.M., Garcia-Dorado D. (2016). Targeting reperfusion injury in patients with ST-segment elevation myocardial infarction: Trials and tribulations. Eur. Heart J..

[52] Onur I., Emet S., Onur S.T., Kara K., Surmen S., Bilge A.K., Adalet K. (2016). PM299 A Novel Parameter for the Diagnosis of Acute Pulmonary Embolism: T Wave Peak to End Interval. Glob. Heart.

[53] Nakagawa T., Yagi T., Ishida A., Mibiki Y., Yamashina Y., Sato H., Sato E., Komatsu J., Saijo Y. (2016). Differences between cardiac memory T wave changes after idiopathic left ventricular tachycardia and ischemic T wave inversion induced by acute coronary syndrome. J. Electrocardiol..

[54] Hermans B.J.M., Bennis F.C., Vink A.S., Koopsen T., Lyon A., Wilde A.A.M., Nuyens D., Robyns T., Pison L., Postema P.G. (2020). Improving long QT syndrome diagnosis by a polynomial-based T-wave morphology characterization. Heart Rhythm.

[55] DeChazal P., O’Dwyer M., Reilly R.B. (2004). Automatic Classification of Heartbeats Using ECG Morphology and Heartbeat Interval Features. IEEE Trans. Biomed. Eng..

[56] Gothwal H., Kedawat S., Kumar R. (2011). Cardiac arrhythmias detection in an ECG beat signal using fast fourier transform and artificial neural network. J. Biomed. Sci. Eng..

[57] Minami K., Nakajima H., Toyoshima T. (1999). Real-time discrimination of ventricular tachyarrhythmia with Fourier-transform neural network. IEEE Trans. Biomed. Eng..

[58] Huang J., Chen B., Yao B., He W. (2019). ECG Arrhythmia Classification Using STFT-Based Spectrogram and Convolutional Neural Network. IEEE Access.

[59] Cohen L. (1989). Time-frequency distributions—A review. Proc. IEEE.

[60] Malfait M., Roose D. (1997). Wavelet-based image denoising using a Markov random field a priori model. IEEE Trans. Image Process..

[61] Torrence C., Compo G.P. (1998). A Practical Guide to Wavelet Analysis. Bull. Am. Meteorol. Soc..

[62] Manikandan M.S., Dandapat S. (2014). Wavelet-based electrocardiogram signal compression methods and their performances: A prospective review. Biomed. Signal Process..

[63] Castillo E., Morales D.P., Botella G., García A., Parrilla L., Palma A.J. (2013). Efficient wavelet-based ECG processing for single-lead FHR extraction. Digit. Signal Process..

[64] Enamamu T., Otebolaku A., Marchang J., Dany J. (2020). Continuous m-Health Data Authentication Using Wavelet Decomposition for Feature Extraction. Sensors.

[65] Li H., Yuan D., Ma X., Cui D., Cao L. (2017). Genetic algorithm for the optimization of features and neural networks in ECG signals classification. Sci. Rep..

[66] Chan H., Siao Y., Chen S., Yu S. (2008). Wavelet-based ECG compression by bit-field preserving and running length encoding. Comput. Meth. Prog. Biomed..

[67] Mazomenos E.B., Biswas D., Acharyya A., Chen T., Maharatna K., Rosengarten J., Morgan J., Curzen N. (2013). A Low-Complexity ECG Feature Extraction Algorithm for Mobile Healthcare Applications. IEEE J. Biomed. Health.

[68] Martis R.J., Acharya U.R., Min L.C. (2013). ECG beat classification using PCA, LDA, ICA and Discrete Wavelet Transform. Biomed. Signal Process..

[69] Liu J., Zhang C., Zhu Y., Ristaniemi T., Parviainen T., Cong F. (2020). Automated detection and localization system of myocardial infarction in single-beat ECG using Dual-Q TQWT and wavelet packet tensor decomposition. Comput. Meth. Prog. Biomed..

[70] Yildirim Ö. (2018). A novel wavelet sequence based on deep bidirectional LSTM network model for ECG signal classification. Comput. Biol. Med..

[71] Jayachandran E.S., Joseph K.P., Acharya U.R. (2010). Analysis of Myocardial Infarction Using Discrete Wavelet Transform. J. Med. Syst..

[72] He H., Tan Y., Xing J. (2019). Unsupervised classification of 12-lead ECG signals using wavelet tensor decomposition and two-dimensional Gaussian spectral clustering. Knowl. Based Syst..

[73] Kumar M., Pachori R.B., Acharya U.R. (2017). Characterization of coronary artery disease using flexible analytic wavelet transform applied on ECG signals. Biomed. Signal Process..

[74] Javadi M., Arani S.A.A.A., Sajedin A., Ebrahimpour R. (2013). Classification of ECG arrhythmia by a modular neural network based on Mixture of Experts and Negatively Correlated Learning. Biomed. Signal Process..

[75] Dilmac S., Korurek M. (2015). ECG heart beat classification method based on modified ABC algorithm. Appl. Soft Comput..

[76] Dima S., Panagiotou C., Mazomenos E.B., Rosengarten J.A., Maharatna K., Gialelis J.V., Curzen N., Morgan J. (2013). On the Detection of Myocadial Scar Based on ECG/VCG Analysis. IEEE Trans. Biomed. Eng..

[77] Kutlu Y., Kuntalp D. (2012). Feature extraction for ECG heartbeats using higher order statistics of WPD coefficients. Comput. Meth. Prog. Biomed..

[78] Tantawi M.M., Revett K., Salem A., Tolba M.F. (2013). Fiducial feature reduction analysis for electrocardiogram (ECG) based biometric recognition. J. Intell. Inf. Syst..

[79] Lee J., McManus D.D., Merchant S., Chon K.H. (2012). Automatic motion and noise artifact detection in Holter ECG data using empirical mode decomposition and statistical approaches. IEEE Trans. Biomed. Eng..

[80] Ince T., Kiranyaz S., Gabbouj M. (2009). A Generic and Robust System for Automated Patient-Specific Classification of ECG Signals. IEEE Trans. Biomed. Eng..

[81] Mar T., Zaunseder S., Martinez J.P., Llamedo M., Poll R. (2011). Optimization of ECG Classification by Means of Feature Selection. IEEE Trans. Biomed. Eng..

[82] de Chazal P., Reilly R.B. (2006). A Patient-Adapting Heartbeat Classifier Using ECG Morphology and Heartbeat Interval Features. IEEE Trans. Biomed. Eng..

[83] Hu Y.H., Palreddy S., Tompkins W.J. (1997). A patient-adaptable ECG beat classifier using a mixture of experts approach. IEEE Trans. Biomed. Eng..

[84] Lee M., Song T., Lee J. (2020). Heartbeat classification using local transform pattern feature and hybrid neural fuzzy-logic system based on self-organizing map. Biomed. Signal Process..

[85] Manikandan M.S., Soman K.P. (2012). A novel method for detecting R-peaks in electrocardiogram (ECG) signal. Biomed. Signal Process..

[86] Zhang Z., Dong J., Luo X., Choi K., Wu X. (2014). Heartbeat classification using disease-specific feature selection. Comput. Biol. Med..

[87] Rodríguez-Sotelo J.L., Cuesta-Frau D., Castellanos-Dominguez G. (2009). Unsupervised classification of atrial heartbeats using a prematurity index and wave morphology features. Med. Biol. Eng. Comput..

[88] Inan O.T., Giovangrandi L., Kovacs G.T.A. (2006). Robust Neural-Network-Based Classification of Premature Ventricular Contractions Using Wavelet Transform and Timing Interval Features. IEEE Trans. Biomed. Eng..

[89] Maršánová L., Ronzhina M., Smíšek R., Vítek M., Němcová A., Smital L., Nováková M. (2017). ECG features and methods for automatic classification of ventricular premature and ischemic heartbeats: A comprehensive experimental study. Sci. Rep..

[90] Elhaj F.A., Salim N., Harris A.R., Swee T.T., Ahmed T. (2016). Arrhythmia recognition and classification using combined linear and nonlinear features of ECG signals. Comput. Meth. Prog. Biomed..

[91] Raj S., Ray K.C. (2018). Sparse representation of ECG signals for automated recognition of cardiac arrhythmias. Exp. Syst. Appl..

[92] Marinho L.B., Nascimento N.D.M.M., Souza J.W.M., Gurgel M.V., Rebouças Filho P.P., de Albuquerque V.H.C. (2019). A novel electrocardiogram feature extraction approach for cardiac arrhythmia classification. Future Gener. Comput. Syst..

[93] Qin Q., Li J., Zhang L., Yue Y., Liu C. (2017). Combining Low-dimensional Wavelet Features and Support Vector Machine for Arrhythmia Beat Classification. Sci. Rep..

[94] Blum A.L., Langley P. (1997). Selection of relevant features and examples in machine learning. Artif. Intell..

[95] Chandrashekar G., Sahin F. (2014). A survey on feature selection methods. Comput. Electr. Eng..

[96] Sufi F., Khalil I. (2011). Faster person identification using compressed ECG in time critical wireless telecardiology applications. J. Netw. Comput. Appl..

[97] Khalaf A.F., Owis M.I., Yassine I.A. (2015). A novel technique for cardiac arrhythmia classification using spectral correlation and support vector machines. Expert Syst. Appl..

[98] Yu S., Chen Y. (2009). Noise-tolerant electrocardiogram beat classification based on higher order statistics of subband components. Artif. Intell. Med..

[99] Llamedo M., Martínez J.P. (2011). Heartbeat Classification Using Feature Selection Driven by Database Generalization Criteria. IEEE Trans. Biomed. Eng..

[100] Kudo M., Sklansky J. (2000). Comparison of algorithms that select features for pattern classifiers. Pattern Recogn..

[101] Wang A., An N., Chen G., Li L., Alterovitz G. (2015). Accelerating wrapper-based feature selection with K-nearest-neighbor. Knowl. Based Syst..

[102] Song C., Liu K., Zhang X., Chen L., Xian X. (2016). An Obstructive Sleep Apnea Detection Approach Using a Discriminative Hidden Markov Model from ECG Signals. IEEE Trans. Biomed. Eng..

[103] Lu M. (2019). Embedded feature selection accounting for unknown data heterogeneity. Exp. Syst. Appl..

[104] Ye C., Kumar B.V., Coimbra M.T. (2012). Heartbeat classification using morphological and dynamic features of ECG signals. IEEE Trans. Biomed. Eng..

[105] He H., Tan Y. (2017). Automatic pattern recognition of ECG signals using entropy-based adaptive dimensionality reduction and clustering. Appl. Soft Comput..

[106] Varatharajan R., Manogaran G., Priyan M.K. (2018). A big data classification approach using LDA with an enhanced SVM method for ECG signals in cloud computing. Multimed. Tools Appl..

[107] Bollmann A., Kanuru N., McTeague K., Walter P., DeLurgio D., Langberg J. (1998). Frequency Analysis of Human Atrial Fibrillation Using the Surface Electrocardiogram and Its Response to Ibutilide. Am. J. Cardiol..

[108] Rieta J.J., Castells F., Sanchez C., Zarzoso V., Millet J. (2004). Atrial Activity Extraction for Atrial Fibrillation Analysis Using Blind Source Separation. IEEE Trans. Biomed. Eng..

[109] Adam M., Oh S.L., Sudarshan V.K., Koh J.E., Hagiwara Y., Tan J.H., San Tan R., Acharya U.R. (2018). Automated characterization of cardiovascular diseases using relative wavelet nonlinear features extracted from ECG signals. Comput. Meth. Prog. Biomed..

[110] Zamani B., Akbari A., Nasersharif B. (2014). Evolutionary combination of kernels for nonlinear feature transformation. Inf. Sci..

[111] Liu F., Zhang W., Gu S. (2016). Local linear Laplacian eigenmaps: A direct extension of LLE. Patt. Recogn. Lett..

[112] Li X., Shu L., Hu H. (2008). Kernel-based nonlinear dimensionality reduction for electrocardiogram recognition. Neural Comput. Appl..

[113] Ayesha S., Hanif M.K., Talib R. (2020). Overview and comparative study of dimensionality reduction techniques for high dimensional data. Inf. Fusion.

[114] Lee J., Reyes B.A., McManus D.D., Maitas O., Chon K.H. (2013). Atrial Fibrillation Detection Using an iPhone 4S. IEEE Trans. Biomed. Eng..

[115] Chen W., Shih J. (2006). A study of Taiwan’s issuer credit rating systems using support vector machines. Exp.Syst. Appl..

[116] Pławiak P. (2018). Novel methodology of cardiac health recognition based on ECG signals and evolutionary-neural system. Exp. Syst. Appl..

[117] Venkatesan C., Karthigaikumar P., Paul A., Satheeskumaran S., Kumar R. (2018). ECG Signal Preprocessing and SVM Classifier-Based Abnormality Detection in Remote Healthcare Applications. IEEE Access.

[118] Raj S., Ray K.C. (2017). ECG Signal Analysis Using DCT-Based DOST and PSO Optimized SVM. IEEE Trans. Instrum. Meas..

[119] Polat K., Güneş S. (2007). Detection of ECG Arrhythmia using a differential expert system approach based on principal component analysis and least square support vector machine. Appl. Math. Comput..

[120] Osowski S., Hoai L.T., Markiewicz T. (2004). Support Vector Machine-Based Expert System for Reliable Heartbeat Recognition. IEEE Trans. Biomed. Eng..

[121] Moavenian M., Khorrami H. (2010). A qualitative comparison of Artificial Neural Networks and Support Vector Machines in ECG arrhythmias classification. Expert Syst. Appl..

[122] Sannino G., De Pietro G. (2018). A deep learning approach for ECG-based heartbeat classification for arrhythmia detection. Future Gener. Comput. Syst..

[123] Goldberger A.L., Amaral L.A.N., Glass L., Hausdorff J.M., Ivanov P.C., Mark R.G., Mietus J.E., Moody G.B., Peng C.-K., Stanley H.E. (2000). PhysioBank, PhysioToolkit, and PhysioNet: Components of a new research resource for complex physiologic signals. Circulation.

[124] Kiranyaz S., Ince T., Gabbouj M. (2016). Real-Time Patient-Specific ECG Classification by 1-D Convolutional Neural Networks. IEEE Trans. Biomed. Eng..

[125] Zhai X., Tin C. (2018). Automated ECG Classification Using Dual Heartbeat Coupling Based on Convolutional Neural Network. IEEE Access.

[126] Tan J.H., Hagiwara Y., Pang W., Lim I., Oh S.L., Adam M., Tan R.S., Chen M., Acharya U.R. (2018). Application of stacked convolutional and long short-term memory network for accurate identification of CAD ECG signals. Comput. Biol. Med..

[127] Acharya U.R., Fujita H., Lih O.S., Adam M., Tan J.H., Chua C.K. (2017). Automated detection of coronary artery disease using different durations of ECG segments with convolutional neural network. Knowl. Based Syst..

[128] Yildirim O., Baloglu U.B., Tan R., Ciaccio E.J., Acharya U.R. (2019). A new approach for arrhythmia classification using deep coded features and LSTM networks. Comput. Meth. Prog. Biomed..

[129] Andersen R.S., Peimankar A., Puthusserypady S. (2019). A deep learning approach for real-time detection of atrial fibrillation. Expert Syst. Appl..

[130] Acharya U.R., Fujita H., Oh S.L., Hagiwara Y., Tan J.H., Adam M. (2017). Application of deep convolutional neural network for automated detection of myocardial infarction using ECG signals. Inf. Sci..

[131] Acharya U.R., Fujita H., Lih O.S., Hagiwara Y., Tan J.H., Adam M. (2017). Automated detection of arrhythmias using different intervals of tachycardia ECG segments with convolutional neural network. Inf. Sci..

[132] Rahhal M.M.A., Bazi Y., AlHichri H., Alajlan N., Melgani F., Yager R.R. (2016). Deep learning approach for active classification of electrocardiogram signals. Inf. Sci..

[133] Shadmand S., Mashoufi B. (2016). A new personalized ECG signal classification algorithm using Block-based Neural Network and Particle Swarm Optimization. Biomed. Signal Process..

[134] Butun E., Yildirim O., Talo M., Tan R., Rajendra Acharya U. (2020). 1D-CADCapsNet: One dimensional deep capsule networks for coronary artery disease detection using ECG signals. Phys. Med..

[135] Baloglu U.B., Talo M., Yildirim O., Tan R.S., Acharya U.R. (2019). Classification of myocardial infarction with multi-lead ECG signals and deep CNN. Pattern Recogn. Lett..

[136] Zhang J., Tian J., Cao Y., Yang Y., Xu X. (2020). Deep time–frequency representation and progressive decision fusion for ECG classification. Knowl. Based Syst..

[137] Poh M., Poh Y.C., Chan P., Wong C., Pun L., Leung W.W., Wong Y., Wong M.M., Chu D.W., Siu C. (2018). Diagnostic assessment of a deep learning system for detecting atrial fibrillation in pulse waveforms. Heart.

[138] Ribeiro A.H., Ribeiro M.H., Paixão G.M.M., Oliveira D.M., Gomes P.R., Canazart J.A., Ferreira M.P.S., Andersson C.R., Macfarlane P.W., Meira W. (2020). Automatic diagnosis of the 12-lead ECG using a deep neural network. Nat. Commun..

[139] Xu S.S., Mak M., Cheung C. (2019). Towards End-to-End ECG Classification with Raw Signal Extraction and Deep Neural Networks. IEEE J. Biomed. Health.

[140] Mahmud T., Fattah S.A., Saquib M. (2020). DeepArrNet: An Efficient Deep CNN Architecture for Automatic Arrhythmia Detection and Classification From Denoised ECG Beats. IEEE Access.

[141] Han C., Shi L. (2020). ML–ResNet: A novel network to detect and locate myocardial infarction using 12 leads ECG. Comput. Meth. Prog. Biomed..

[142] Yıldırım Ö., Pławiak P., Tan R., Acharya U.R. (2018). Arrhythmia detection using deep convolutional neural network with long duration ECG signals. Comput. Biol. Med..

[143] Carrera D., Rossi B., Fragneto P., Boracchi G. (2019). Online anomaly detection for long-term ECG monitoring using wearable devices. Pattern Recogn..

[144] Attia Z.I., Kapa S., Lopez-Jimenez F., McKie P.M., Ladewig D.J., Satam G., Pellikka P.A., Enriquez-Sarano M., Noseworthy P.A., Munger T.M. (2019). Screening for cardiac contractile dysfunction using an artificial intelligence–enabled electrocardiogram. Nat. Med..

[145] Buja L.M. (2015). Coronary Artery Disease: Pathological Anatomy and Pathogenesis. Coronary Artery Disease.

[146] Acharya U.R., Sudarshan V.K., Koh J.E.W., Martis R.J., Tan J.H., Oh S.L., Muhammad A., Hagiwara Y., Mookiah M.R.K., Chua K.P. (2017). Application of higher-order spectra for the characterization of Coronary artery disease using electrocardiogram signals. Biomed. Signal Process..

[147] Patidar S., Pachori R.B., Rajendra Acharya U. (2015). Automated diagnosis of coronary artery disease using tunable-Q wavelet transform applied on heart rate signals. Knowl. Based Syst..

[148] Acharya U.R., Fujita H., Sudarshan V.K., Oh S.L., Adam M., Tan J.H., Koo J.H., Jain A., Lim C.M., Chua K.C. (2017). Automated characterization of coronary artery disease, myocardial infarction, and congestive heart failure using contourlet and shearlet transforms of electrocardiogram signal. Knowl. Based Syst..

[149] De Albuquerque V.H.C., Nunes T.M., Pereira D.R., Luz E.J.D.S., Menotti D., Papa J.P., Tavares J.M.R.S. (2018). Robust automated cardiac arrhythmia detection in ECG beat signals. Neural Comput. Appl..

[150] Sangaiah A.K., Arumugam M., Bian G. (2020). An intelligent learning approach for improving ECG signal classification and arrhythmia analysis. Artif. Intell. Med..

[151] Romdhane T.F., Alhichri H., Ouni R., Atri M. (2020). Electrocardiogram heartbeat classification based on a deep convolutional neural network and focal loss. Comput. Biol. Med..

[152] Stern S., Sclarowsky S. (2009). The ECG in Diabetes Mellitus. Circulation.

[153] Ben Halima M., Boudiche S., Sammoud K., Ben Amar J., Rekik B., Larbi N., Ouali S., Farhati A., Aouina H., Mghaieth F. (2020). Severe obstructive sleep apnea in Tunisian population with atrial fibrillation: Prevalence and predictive factors. Arch. Cardiovasc. Dis. Suppl..

[154] Goldman A., Hod H., Chetrit A., Dankner R. (2019). Incidental abnormal ECG findings and long-term cardiovascular morbidity and all-cause mortality: A population based prospective study. Int. J. Cardiol..

[155] Sengupta P.P., Kulkarni H., Narula J. (2018). Prediction of Abnormal Myocardial Relaxation from Signal Processed Surface ECG. J. Am. Coll. Cardiol..

[156] Cui X., Chang H., Lin L., Yu C., Hsieh W., Li W., Peng C., Lin J., Lo M. (2019). Prediction of atrial fibrillation recurrence before catheter ablation using an adaptive nonlinear and non-stationary surface ECG analysis. Phys. A Stat. Mech. ITS Appl..

[157] Liao Y., Chung F., Lin Y., Chang S., Lo L., Hu Y., Tuan T., Chao T., Liao J., Lin C. (2017). The application of signal average ECG in the prediction of recurrences after catheter ablation of ventricular arrhythmias in arrhythmogenic right ventricular dysplasia/cardiomyopathy. Int. J. Cardiol..

[158] Vafaie M.H., Ataei M., Koofigar H.R. (2014). Heart diseases prediction based on ECG signals’ classification using a genetic-fuzzy system and dynamical model of ECG signals. Biomed. Signal Process..

[159] Venkatesan C., Karthigaikumar P., Satheeskumaran S. (2018). Mobile cloud computing for ECG telemonitoring and real-time coronary heart disease risk detection. Biomed. Signal Process..

[160] Piwek L., Ellis D.A., Andrews S., Joinson A. (2016). The Rise of Consumer Health Wearables: Promises and Barriers. PLoS Med..

[161] Friberg L., Bergfeldt L. (2013). Atrial fibrillation prevalence revisited. J. Intern. Med..

[162] Camm A.J., Corbucci G., Padeletti L. (2012). Usefulness of Continuous Electrocardiographic Monitoring for Atrial Fibrillation. Am. J. Cardiol..

[163] Aronsson M., Svennberg E., Rosenqvist M., Engdahl J., Al-Khalili F., Friberg L., Frykman-Kull V., Levin L. (2015). Cost-effectiveness of mass screening for untreated atrial fibrillation using intermittent ECG recording. Europace.

[164] Svennberg E., Engdahl J., Al-Khalili F., Friberg L., Frykman V., Rosenqvist M. (2015). Mass Screening for Untreated Atrial Fibrillation. Circulation.

[165] Elgendi M., Eskofier B., Dokos S., Abbott D. (2014). Revisiting QRS Detection Methodologies for Portable, Wearable, Battery-Operated, and Wireless ECG Systems. PLoS ONE.

[166] Elgendi M., Mohamed A., Ward R. (2017). Efficient ECG Compression and QRS Detection for E-Health Applications. Sci. Rep..

[167] Majumder S., Chen L., Marinov O., Chen C., Mondal T., Deen M.J. (2018). Noncontact Wearable Wireless ECG Systems for Long-Term Monitoring. IEEE Rev. Biomed. Eng..

[168] Lee J.S., Lee S.J., Choi M., Seo M., Kim S.W. (2019). QRS detection method based on fully convolutional networks for capacitive electrocardiogram. Exp. Syst. Appl..

[169] Yousefi R., Nourani M., Ostadabbas S., Panahi I. (2014). A Motion-Tolerant Adaptive Algorithm for Wearable Photoplethysmographic Biosensors. IEEE J. Biomed. Health.

[170] Pereira T., Tran N., Gadhoumi K., Pelter M.M., Do D.H., Lee R.J., Colorado R., Meisel K., Hu X. (2020). Photoplethysmography based atrial fibrillation detection: A review. NPJ Digit. Med..

[171] Zhu Q., Tian X., Wong C., Wu M. (2019). Learning Your Heart Actions From Pulse: ECG Waveform Reconstruction From PPG. bioRxiv.

[172] Vandenberk T., Stans J., Mortelmans C., Van Haelst R., Van Schelvergem G., Pelckmans C., Smeets C.J., Lanssens D., De Cannière H., Storms V. (2017). Clinical Validation of Heart Rate Apps: Mixed-Methods Evaluation Study. JMIR Mhealth Uhealth.

[173] Rundo F., Conoci S., Ortis A., Battiato S. (2018). An Advanced Bio-Inspired PhotoPlethysmoGraphy (PPG) and ECG Pattern Recognition System for Medical Assessment. Sensors.

[174] Elgendi M., Fletcher R., Liang Y., Howard N., Lovell N.H., Abbott D., Lim K., Ward R. (2019). The use of photoplethysmography for assessing hypertension. NPJ Digit. Med..

[175] Bumgarner J.M., Lambert C.T., Hussein A.A., Cantillon D.J., Baranowski B., Wolski K., Lindsay B.D., Wazni O.M., Tarakji K.G. (2018). Smartwatch Algorithm for Automated Detection of Atrial Fibrillation. J. Am. Coll. Cardiol..

[176] Lau J.K., Lowres N., Neubeck L., Brieger D.B., Sy R.W., Galloway C.D., Albert D.E., Freedman S.B. (2013). iPhone ECG application for community screening to detect silent atrial fibrillation: A novel technology to prevent stroke. Int. J. Cardiol..

[177] Chon K.H., McManus D.D. (2018). Detection of atrial fibrillation using a smartwatch. Nat. Rev. Cardiol..

[178] Ganesan A.N., Chew D.P., Hartshorne T., Selvanayagam J.B., Aylward P.E., Sanders P., McGavigan A.D. (2016). The impact of atrial fibrillation type on the risk of thromboembolism, mortality, and bleeding: A systematic review and meta-analysis. Eur. Heart J..

[179] Walch O., Huang Y., Forger D., Goldstein C. (2019). Sleep stage prediction with raw acceleration and photoplethysmography heart rate data derived from a consumer wearable device. Sleep.

[180] Zubair M., Yoon C. (2020). Multilevel mental stress detection using ultra-short pulse rate variability series. Biomed. Signal Process..

